# CellectSeq: In silico discovery of antibodies targeting integral membrane proteins combining in situ selections and next-generation sequencing

**DOI:** 10.1038/s42003-021-02066-5

**Published:** 2021-05-12

**Authors:** Abdellali Kelil, Eugenio Gallo, Sunandan Banerjee, Jarrett J. Adams, Sachdev S. Sidhu

**Affiliations:** 1 Donnelly Centre for Cellular and Biomolecular Research, University of Toronto, Toronto, Canada; 2 Toronto Recombinant Antibody Centre, University of Toronto, Toronto, Canada

**Keywords:** Statistical methods, Bioinformatics, High-throughput screening, Sequencing, Antibody therapy

## Abstract

Synthetic antibody (Ab) technologies are efficient and cost-effective platforms for the generation of monoclonal Abs against human antigens. Yet, they typically depend on purified proteins, which exclude integral membrane proteins that require the lipid bilayers to support their native structure and function. Here, we present an Ab discovery strategy, termed CellectSeq, for targeting integral membrane proteins on native cells in complex environment. As proof of concept, we targeted three transmembrane proteins linked to cancer, tetraspanin CD151, carbonic anhydrase 9, and integrin-α11. First, we performed in situ cell-based selections to enrich phage-displayed synthetic Ab pools for antigen-specific binders. Then, we designed next-generation sequencing procedures to explore Ab diversities and abundances. Finally, we developed motif-based scoring and sequencing error-filtering algorithms for the comprehensive interrogation of next-generation sequencing pools to identify Abs with high diversities and specificities, even at extremely low abundances, which are very difficult to identify using manual sampling or sequence abundances.

## Abstract (web summary)

Kelil, Gallo et al. develop an antibody discovery strategy, termed CellectSeq, for targeting integral membrane proteins on native cells. This strategy uses motif-based scoring and sequencing error-filtering algorithms to identify antibodies with high diversity and specificity even at extremely low abundances in NGS pools.

## Introduction

The application of antibodies (Abs)^1^ for targeting cell surface proteins has prompted the development of synthetic human Abs^2^. These are constructed from scratch using designed synthetic DNA that allows for precise control over design. Therefore, synthetic Abs utilize highly optimized human frameworks that introduce defined chemical diversity at positions most likely to contribute to antigen recognition (see Methods). By this method, synthetic phage-displayed libraries containing >10^10^ unique Abs can be constructed to rival the combinatorial diversity of natural in vivo immune repertoires and, in many ways, may outperform natural repertoires for the production of Abs with high affinities and specificities^2,3^. Moreover, synthetic as well as immune-based Ab phage display technologies have also proven amenable to automation to enable high-throughput methods of selection to target large families of soluble antigens^4–6^. Altogether, phage display provides a rapid and effective strategy for isolating Abs against target antigens.

However, a major limitation to both in vitro and in vivo methods for antibody generation is the difficulty of targeting multi-pass integral membrane proteins, which generally cannot be purified in a native form in the absence of the cell membrane. Integral membrane proteins remain a recalcitrant group of critical targets for Ab development due to their inherent association with the lipid bilayer, differential multi-conformational states^7,8^, and interactions with other cell surface proteins^9,10^. Moreover, multi-pass integral membrane proteins often lack large, structured domains in their extracellular regions^11,12^, and thus, pose a particular challenge for recombinant expression and purification^13^. Given that many essential biological processes and diseases depend on integral membrane proteins, the difficulties in targeting this large subset of the human proteome are a major roadblock in many areas of biological research and drug development^14,15^.

With 33 members in the human proteome, the tetraspanin receptor family (Pfam:PF00335, Transmembrane 4 superfamily) represents a particularly interesting set of potential therapeutic targets, as many family members are involved in processes implicated in cancer progression, including tumor proliferation, migration, and metastasis^16–18^. In particular, cluster of differentiation 151^19–23^ (CD151; Fig. S1), also known as PETA-3 or SFA-1, is a 30 kD tetraspanin receptor that is widely expressed in normal cells and tissues (e.g., epithelium, endothelium, cardiac muscle, dendritic cells, and hematopoietic cells)^24^, and overexpressed in diverse tumor tissues (e.g., lung, colon, prostate, pancreas, breast, and skin)^25–27^. Moreover, the elevated expression of CD151 is correlated with cancer patient mortality and enhanced metastasis of tumors^28,29^. The primary role of CD151 in cancer appears to be its ability to organize the distribution and function of growth factor receptors and integrins^25,30^. Consequently, CD151 may guide the migratory activity of tumor cells to induce invasiveness and metastasis. CD151 also modulates the pharmacological response of therapeutics that antagonize other cell surface receptors^31^, and appears to synergize and modulate intracellular signal activities in cancer. For example, integrin-associated CD151 may drive HER2 evoked mammary tumor onset and metastasis and may enhance the activation of HER2 and other receptor tyrosine kinases by regulating dimerization^32–34^. Thus, CD151 is an integral membrane protein that may be a promising therapeutic target for the development of Abs that can antagonize the interactions mediated by its extracellular domains. However, CD151 receptor extracellular portion consists of only two peptides of 15 and 104 amino acids^35^, which makes it a challenging target for antibody selection (Fig. S1).

Recently, we reported optimized methods for in situ selections with phage-displayed synthetic antibody libraries with native antigen on live cells to develop a large panel of selective Abs for integrin-α11/β1, a marker of aggressive tumors that is involved in stroma-tumor crosstalk^36^. Manual screening of over one thousand phage clones identified unique Abs with strong and selective binding to cells expressing integrin-α11/β1, and notably, most of these Abs did not recognize the purified antigen, suggesting that cell-based selections were essential for targeting native epitopes^36^. Moreover, next generation sequencing (NGS) analysis of the abundance of unique clones in the selection pools showed that most of the Abs identified by clonal screening were among the most abundant and enriched amongst the NGS sequences, but intriguingly, many other sequences were also identified, suggesting that the clonal screening had only isolated a small subset of antigen-specific clones^36^.

**Fig. 1:**
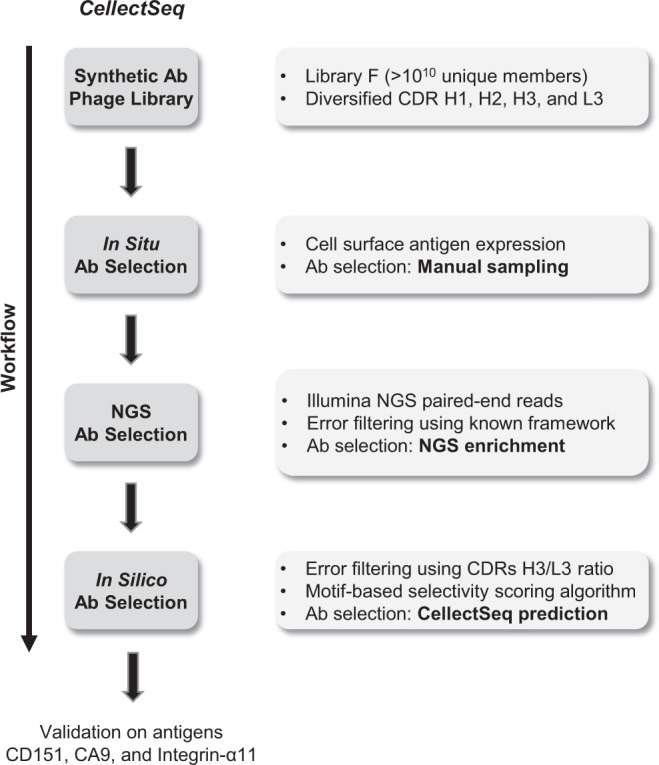
Flow-chart of workflow for the CellectSeq process. Manual sampling: Candidate clones were selected manually from round four phage output positive pool. NGS enrichment ranking: Candidate clones were selected from NGS output based on (i) their abundance in the positive pool and (ii) their enrichment in the positive compared to the negative pool, precisely, they were taken from the top-right quadrant in Fig. 3. CellectSeq prediction: Candidate clones were predicted from NGS output using the motif-based algorithm with *p* values < 10^−10^.

Here, we performed in situ cell-based selection procedures to enrich Ab-phage pools for antigen-specific binders to CD151. By means of NGS we implemented an in silico analysis to efficiently explore and identify unique antigen-specific clones for CD151 in the enriched pools. In conjunction with commercial gene synthesis and recombinant Ab production strategies, the antigen-specific Ab-phage sequences were purified as Abs for direct assessment of cell-surface antigen recognition. We have collectively termed this methodology “CellectSeq” (Fig. 1), which allows the comprehensive interrogation of candidate Abs in enriched but highly diverse Ab-phage pools. To prove the advantage of CellectSeq, we manually isolated 96 random Ab-phage clones and identified one immunodominant clone as a binder for CD151 using cellular phage ELISA^37^. We then applied NGS enrichment ranking which yielded 100 clones with more than 200 counts in the positive pool and more than fourfold enrichment relative to the negative pool, but all were close homologs of the immunodominant clone found earlier. Next, we applied the in silico strategy which identified four clones with high diversity including the immunodominant clone, with *p* values below 10^−10^ and frequencies varying from very high (30%) to as low as one in a million sequence reads. The four clones identified by CellectSeq were proven highly specific using quantitative flow cytometry and immunoprecipitation mass spectrometry (IP-MS).

We further validated CellectSeq on two distinct cell-surface receptors. We targeted carbonic anhydrase 9 (CA9), an enzyme associated with remodeling the tumor pH environment^38,39^, and integrin-α11β1, a heterodimeric receptor involved in cancer-associated fibroblast biology^36,40,41^. Taken together, we show that CellectSeq can identify rare but highly selective and diverse Abs targeting integral membrane proteins, without the need for screening of individual clones at the phage level. The technology should be applicable for the generation of Abs targeting many integral membrane proteins that have proven recalcitrant to conventional in vivo and in vitro methods.

## Results

### Features of synthetic library F

To generate Abs targeting cell surface receptors, we used the phage-displayed Library F^42^ of synthetic antigen-binding fragments (Fabs) that offers advantageous features for CellectSeq (Fig. S2). First, Library F is extremely diverse (>10^10^ unique members) and precisely designed to ensure that most members are stable and well-displayed on phage^43^. Second, the library have proven functional for selections with either purified antigens^42^ or cell-surface antigens^36^ and has yielded numerous selective Abs^36^. Third, the library was constructed with a single, highly stable human framework resulting in negligible display bias, where most library members are presented at similar levels^42^. Also, as previously observed in similar libraries, the abundances of individual clones in pools enriched for target antigens are correlated with relative affinities;^44^ this property is important to enhance NGS analysis based on enrichment ranking, allowing for the identification of clones with high affinity and selectivity. Fourth, the synthetic Abs are diversified at only four complementary determining regions (CDRs; H1, H2 and H3, and L3), which permit standard NGS procedures utilizing primers that anneal to common framework regions in a cost-effective manner (Figs. S3 and S4). Fifth, each of the four CDRs is composed of defined amino acid positions with restricted diversities. Therefore, the NGS data quality can be very accurately evaluated by assessing any deviations from the fixed framework or occurrence of unexpected codons at diversified positions. For instance, CDRs H1 and H2 contain only six or eight binary degenerate codons and contain a diversity of 64 and 256 unique sequences, respectively (Fig. S2D). Conversely, CDRs L3 and H3 are much more diverse in terms of loop lengths (3–7 or 1–17 degenerate codons, respectively), and in terms of sequence composition (encoded by defined ratios of nine codons encoding nine amino acids). The CDRs L3 and H3 offer a theoretical diversity of the order of 10^9^ and 10^16^ unique sequences, respectively (Fig. S2D**)**. Ultimately, the four CDRs combined provide a practical diversity of >10^10^ unique clones^42^. Thus, the highly diverse Library F, with defined length and chemical diversity encoded in CDRs L3, H1, H2, and H3, permits the precise probabilistic detection and elimination of artifactual CDR sequence combinations from NGS data, such as those derived from PCR sequence amplifications required for the NGS process^45^ (see Methods).

### Cell-based in situ selection for anti-CD151 Abs

We performed in situ selections against cell-surface CD151 on live cells, where CD151 is targeted in its native cell-surface environment. For cell engineering, we selected the HEK293T cell line because it grows rapidly and exhibits high display of transgenic cell surface proteins^46^. To enrich binders for CD151, we engineered the HEK293T cells to stably overexpress CD151 (HEK293T-CD151+; positive cells) (Fig. S5A). Conversely, to deplete non-target-selective binders we engineered HEK293T cells that stably expressed a short hair-pin RNA that depleted CD151 mRNA, and consequently, reduced cell-surface display of CD151 (HEK293T-CD151^−^; negative cells) (Fig. S5A). The strategy of CellectSeq in situ selections utilizes multiple rounds of selection against antigen positive and negative cells, where it aims to produce a positive Ab pool enriched with selective clones for the target antigen, and a negative Ab pool enriched with non-specific clones (background).

**Fig. 2:**
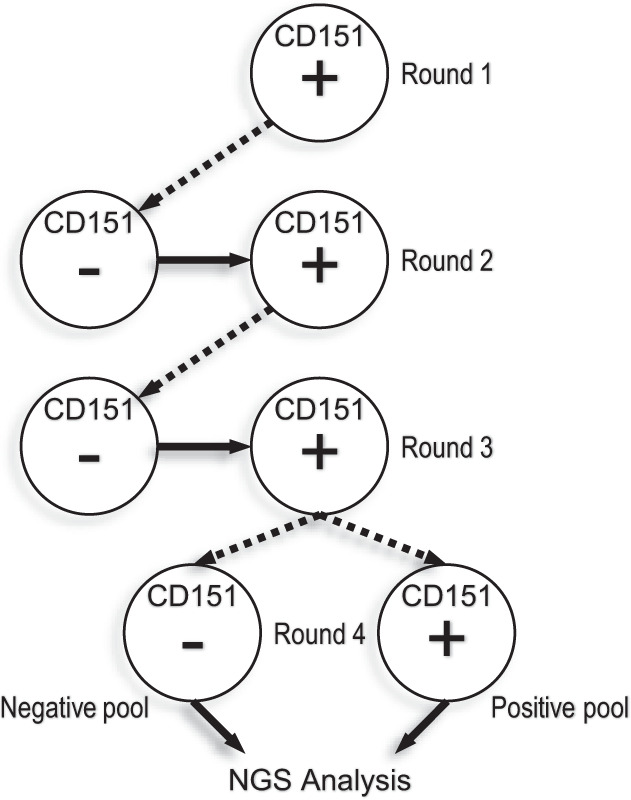
CD151 in situ Ab selection process. Circles represent live HEK293T cell lines of either overexpressing CD151 (HEK293T-CD151+; denoted by “+” sign), or CD151 knockdown cells (HEK293T-CD151−; denoted by a “−” sign). For round 1, the Fab-phage (naïve library F) were incubated with HEK293T-CD151+ cells, then eluted and amplified for the next round. For rounds 2 and 3, the Fab-phage were first incubated with HEK293T-CD151− cells, then transferred and incubated with HEK293T-CD151+ cells. For round 4, amplified round 3 Fab-phage were independently incubated with HEK293T-CD151− or HEK293T-CD151+ cells. The dashed lines with arrows indicate that eluted phage from the preceding round were amplified through *E. coli* prior to incubation with cells in the succeeding round. The solid lines with arrows indicate that unbound phage from the preceding cell line were transferred directly to the succeeding cell line. In round 4, phages from Fab-phage pools HEK293T-CD151+ (Positive pool) and HEK293T-CD151− (Negative pool) were amplified and used as DNA template for NGS analysis.

To this end, the naïve phage pool representing Library F was subjected to four rounds of selections with the engineered cell lines (Fig. 2). Round 1 consisted of a positive selection on HEK293T-CD151+ cells to enrich for Fab-phage that bound to CD151, followed by elution of bound phage and amplification by passage through *E. coli*. In round 2, we employed a strategy whereby phage pools were exposed to control cells HEK293T-CD151− to deplete clones that bound to other cell-surface antigens, followed by positive selections with HEK293T-CD151+ cells. Round 3 repeated the round 2 process using the amplified phage pool from round 2. For the last round, the amplified phage pool from round 3 was split into two pools, and then subjected to a round 4 selection process that involved elution and amplification of phage bound to either HEK293T-CD151+ cells (positive selection) or to HEK293T-CD151− cells (negative selection) (Fig. 2). Thus, the round 4 phage selection output consisted of two pools, a positive and a negative pool.

After the four rounds of selection for binding to in situ CD151, we manually isolated 96 random Ab-phage clones derived from the round 4 phage output of HEK293T-CD151+ cells (positive pool). We screened all 96 clones by cellular phage ELISA^37^, where phage signals were measured for binding to HEK293T-CD151+ cells and compared to control HEK293T-CD151− cells. Here, we identified 49 phage clones, with binding signals fivefold or greater over controls deemed as positive binders for cellular CD151 (Fig. S6). The remaining clones were negative clones with low binding signals to both cell types and non-specific clones that bound to both cell types. After Sanger DNA sequencing analysis, all 49 clones shared the same sequence of clone CD151-1 (Table 1), indicating the Ab selection enriched for an immune-dominant clone. Thus, manual Ab screening failed at deriving multiple unique and diversified CD151 selective clones, and consequently, we next performed NGS analysis of the output selection.

**Table 1:** Ab clones derived from CellectSeq.

Clone ID	NGS Pos pool		Log10 FC Pos/Neg	*P* value	Effect size	EC50 (nM)	L3									H1						H2										H3																	
	Count	% total					107	108	109	110	111	113	114	115	116	30	35	36	37	38	39	55	56	57	58	59	62	63	64	65	66	107	108	109	110	111	111.1	111.2	111.3	111.4	111.5	112.4	112.3	112.2	112.1	112	113	114	115
CD151-1	226048	3.0E-01	2.17	2.2E-303	3.1E+2	25	S	F	F	-	-	-	-	P	I	L	S	Y	Y	S	M	S	I	Y	P	S	Y	G	Y	T	Y	S	H	Y	G	V	-	-	-	-	-	-	-	-	W	Y	G	A	M
CD151-2	137	1.8E-05	1.26	8.0E-130	2.9E+0	71	S	W	V	Y	-	-	S	L	I	I	Y	Y	Y	S	M	S	I	S	P	Y	S	G	Y	T	Y	S	P	Y	A	V	-	-	-	-	-	-	-	-	-	F	Y	G	M
CD151-3	74	9.8E-06	1.72	7.9E-294	1.6E+0	36	S	G	W	P	-	-	F	L	I	L	S	Y	Y	Y	M	S	I	Y	P	Y	Y	G	Y	T	Y	S	H	Y	G	V	-	-	-	-	-	-	-	-	W	Y	G	A	M
CD151-4	7	9.3E-07	0.54	3.1E-24	1.2E+0	177	S	S	Y	-	-	-	S	L	I	L	S	Y	S	Y	M	S	I	S	S	S	Y	G	Y	T	S	S	Y	S	G	-	-	-	-	-	-	-	-	-	-	-	G	A	L
CA9-01	1613	9.0E-04	1.7	6.8E-270	1.8E+0		G	P	Y	-	-	-	G	L	I	L	S	Y	Y	S	I	S	I	S	S	Y	Y	G	Y	T	S	G	G	W	-	-	-	-	-	-	-	-	-	-	-	-	Y	G	L
CA9-02	214	1.2E-04	1.0	1.7E-17	3.4E+0		S	G	Y	V	-	-	Y	P	I	L	S	Y	Y	S	I	S	I	S	S	Y	Y	S	Y	T	S	V	G	-	-	-	-	-	-	-	-	-	-	-	-	-	-	-	F
CA9-03	2780	1.5E-03	0.5	5.0E-21	3.4E+1		S	Y	S	-	-	-	-	L	I	L	S	Y	Y	S	I	S	I	Y	S	Y	Y	G	Y	T	S	W	P	S	F	Y	-	-	-	-	-	-	-	-	Y	V	P	A	M
CA9-04	28223	1.6E-02	0.8	5.7E-303	8.6E+2		G	G	S	H	-	-	S	P	I	L	S	Y	Y	S	M	S	I	S	S	Y	Y	G	Y	T	Y	S	Y	P	A	S	G	-	-	-	-	-	-	W	W	H	Y	A	I
CA9-05	19266	1.1E-02	2.0	1.5E-15	1.7E+3		Y	S	H	Y	S	H	Y	P	I	L	S	Y	Y	S	M	S	I	Y	P	Y	Y	G	Y	T	Y	Y	G	W	G	H	G	W	Y	-	-	-	-	F	S	V	A	G	F
CA9-06	8027	4.5E-03	1.6	4.6E-47	3.6E+1		P	G	Y	-	-	-	A	P	I	I	S	Y	Y	S	I	S	I	S	S	Y	Y	G	Y	T	S	G	G	W	-	-	-	-	-	-	-	-	-	-	-	-	Y	G	L
CA9-07	499	2.8E-04	0.8	1.2E-281	1.6E+1		S	S	Y	-	-	-	S	L	I	I	S	Y	S	S	M	S	I	Y	S	Y	Y	G	S	T	S	Y	V	P	F	Y	S	-	-	-	-	-	-	-	H	W	V	A	L
CA9-08	9	5.0E-06	INF	2.3E-186	1.3E+0		Y	S	Y	-	-	-	Y	P	I	I	S	Y	Y	S	I	S	I	S	S	Y	Y	S	Y	T	S	S	G	-	-	-	-	-	-	-	-	-	-	-	-	-	-	-	F
CA9-09	16	8.9E-06	INF	1.6E-179	3.8E+0		S	S	Y	-	-	-	S	L	I	L	S	Y	Y	S	I	S	I	Y	P	Y	Y	S	S	T	S	G	Y	W	W	Y	G	S	-	-	-	-	-	H	W	Y	S	G	M
CA9-10	45	2.5E-05	0.6	2.2E-246	2.6E+0		S	S	Y	-	-	-	S	L	I	I	S	Y	Y	S	M	S	I	Y	S	Y	Y	G	S	T	S	H	Y	W	P	W	Y	-	-	-	-	-	-	-	Y	S	G	A	M
CA9-11	8	4.5E-06	INF	1.9E-41	1.3E+0		G	S	Y	-	-	-	Y	P	I	I	S	Y	Y	S	I	S	I	S	S	Y	Y	S	Y	T	S	A	G	-	-	-	-	-	-	-	-	-	-	-	-	-	-	-	F
CA9-12	52	2.9E-05	1.5	6.1E-13	1.3E+0		S	S	S	-	-	-	Y	L	F	I	S	Y	Y	S	M	S	I	S	S	Y	S	G	Y	T	S	Y	P	S	F	Y	-	-	-	-	-	-	-	-	Y	A	P	G	L
A11-S1	135355	3.0E-01	2.0	2.4E-303	1.4E+2		Y	G	W	-	-	-	-	L	I	I	S	Y	Y	Y	M	S	I	Y	P	S	S	S	Y	T	Y	Y	S	Y	S	-	-	-	-	-	-	-	-	-	-	H	Y	G	M
A11-S2	5640	1.3E-03	1.8	1.1E-11	6.8E+1		S	F	H	-	-	-	Y	L	F	I	S	Y	S	S	M	S	I	S	P	Y	S	G	S	T	Y	S	Y	-	-	-	-	-	-	-	-	-	-	-	-	-	-	A	L
A11-S3	4001	9.0E-04	2.5	2.4E-303	9.1E+0		Y	G	W	-	-	-	-	L	I	L	S	Y	S	S	M	S	I	S	P	Y	S	G	Y	T	Y	Y	V	G	-	-	-	-	-	-	-	-	-	-	-	-	V	G	F
A11-S4	6	1.4E-06	INF	5.2E-149	1.2E+0		Y	G	W	-	-	-	-	L	I	I	Y	Y	Y	S	M	S	I	S	P	Y	Y	G	Y	T	Y	G	Y	Y	W	-	-	-	-	-	-	-	-	-	-	-	G	G	L
A11-S5	12369	2.8E-03	1.5	2.4E-303	7.3E+2		S	Y	W	-	-	-	Y	L	F	I	S	Y	S	S	M	Y	I	Y	P	Y	Y	G	Y	T	Y	G	S	W	S	V	-	-	-	-	-	-	-	-	-	H	P	A	M
A11-S6	135	3.0E-05	INF	9.2E-16	3.9E+0		S	G	P	Y	-	S	S	L	F	L	S	Y	S	S	M	Y	I	Y	P	S	S	G	Y	T	S	G	Y	F	W	S	S	W	H	-	-	H	S	Y	Y	G	A	A	M
A11-N1	129366	2.9E-02	−0.1	5.0E-02	1.7E-2		Y	W	Y	W	-	Y	Y	L	F	L	Y	S	S	S	M	Y	I	Y	P	Y	Y	G	Y	T	Y	G	Y	Y	W	-	-	-	-	-	-	-	-	-	-	-	G	G	L
A11-N2	35490	8.0E-03	−0.2	7.0E-03	2.3E-2		G	G	Y	W	-	G	Y	P	I	I	Y	Y	S	S	M	S	I	Y	P	S	S	G	Y	T	Y	Y	Y	S	V	-	-	-	-	-	-	-	-	-	-	-	Y	A	M
A11-N3	82968	1.9E-02	−0.3	9.0E-03	3.5E-1		S	Y	P	S	-	S	Y	L	F	I	Y	Y	S	Y	M	S	I	Y	S	Y	S	G	Y	T	S	S	F	S	G	W	S	S	V	-	P	S	S	G	Y	W	Y	A	L
A11-N4	26483	6.0E-03	−1.0	9.0E-01	8.0E-3		Y	W	W	H	-	G	F	L	I	L	Y	Y	S	S	M	S	I	Y	P	Y	Y	G	Y	T	S	S	G	P	S	G	-	-	-	-	-	-	-	-	G	Y	G	G	L
A11-N5	2295	5.2E-04	0.0	6.0E-02	1.4E-2		F	W	G	-	-	-	-	P	I	F	S	S	S	S	I	S	I	S	S	S	Y	G	Y	T	Y	S	G	Y	P	Y	G	S	W	Y	G	Y	W	W	Y	P	Y	A	M
A11-N6	343974	7.7E-02	−0.7	4.0E-01	2.8E-1		W	S	Y	-	-	-	S	L	F	I	Y	Y	Y	S	M	S	I	S	P	Y	Y	S	Y	T	S	S	Y	H	Y	-	-	-	-	-	-	-	-	-	-	G	W	G	F

Summary of identified clones showing the NGS unique sequence counts, abundance (% total), and the enrichment in the positive pool versus the negative pool, the obtained *p* value using the motif-based method and the corresponding effect size (Cohen’s index), the Ab concentrations that result in half-maximal affinities (EC50) for CD151, and the sequence of amino acids in the diversified CDR loop regions, as defined by the IMGT nomenclature^77^. The expression INF (infinity) refers to zero observations in the negative pool.

### NGS enrichment ranking selection for anti-CD151 Abs

To identify unique CD151 specific Fab-phage clones in the round 4 selection output, we performed NGS analysis to explore the output diversity and relative abundance of every Ab clone. Therefore, we deep sequenced the round 4 output derived from the positive and negative pools. This allowed us to obtain CD151 selective sequences (derived from the positive pool), and non-specific background sequences (derived from the negative pool). The phage DNA from the Ab selection output pools were subjected to PCR amplification resulting in amplicons with Illumina NGS adapter sequences and unique barcode identifiers that flanked the region of CDRs L3 and H3 (Figs. S3 and S4). The amplicons from each output pool (positive and negative) were quality controlled for correct size, purified, and quantified, then normalized and pooled, and finally sequenced using an Illumina HiSeq 2500 instrument (see Methods). Besides the Illumina universal sequencing primers (PE1 and PE2), the NGS runs also included a custom primer that allowed for the complete sequencing of CDRs H1 and H2. Thus, the three primer reads (PE1, PE2, and custom; Figs. S3 and S4) provided the complete sequence coverage of the four diversified CDRs in Library F^42^ (Fig. S2). We performed duplicate NGS runs, and each run controlled for high sequence quality scores^47,48^, then merged to maximize frequencies and numbers of unique sequences (see Methods). The sequences were filtered from instrument sequencing errors using per base high quality score cut-off of *Q* = 30, which corresponds to 1:1000 of incorrect base call^43^. Following, all sequencing reads from the duplicate NGS runs were combined and deconvoluted. The three different primer reads (PE1, PE2, and custom) for each clone were assembled into a single sequence to derive the complete synthetic Ab sequence (see Methods).

To remove technical errors inherent to Illumina sequencing and PCR amplification, we compared the assembled sequences to the theoretical sequence repertoires of Library F^42^. For each Ab clone, the nucleotide sequences were evaluated for codon deviations from the synthetic design of the fixed framework and restricted CDR positions (Figure S2A, B). Any divergent sequences from the synthetic library were discarded. Subsequently, the sequences were filtered for potential PCR-induced artifacts that may arise during the NGS sample preparation and Illumina sequencing process (see Methods). These may occur due to incorrect annealing amalgams (i.e., combinations) of different clones^45^, which for our case may be driven by the fixed Ab framework coding region. Therefore, for both pools, for every sequence we calculated the frequencies (i.e., number of observations) of CDRs H3 and L3, respectively, since these two CDRs are the most diversified in the synthetic library (Fig. S2D) and drive the majority of affinity interactions with the antigen^42^. We then identified statistically valid L3/H3 pairs by calculating a frequency cut-off to determine a minimal threshold of valid occurrences, with all below-threshold pairs filtered from the selection pool (see Methods). Thus, we obtained 7,541,189 and 7,250,873 high quality NGS reads for the positive and negative pool, respectively. The reads were then translated into amino acid. This process ultimately yielded 105,434 (Supplementary Data 1) and 251,621 (Supplementary Data 2) unique amino acid sequences in the positive and negative pools, respectively.

**Fig. 3:**
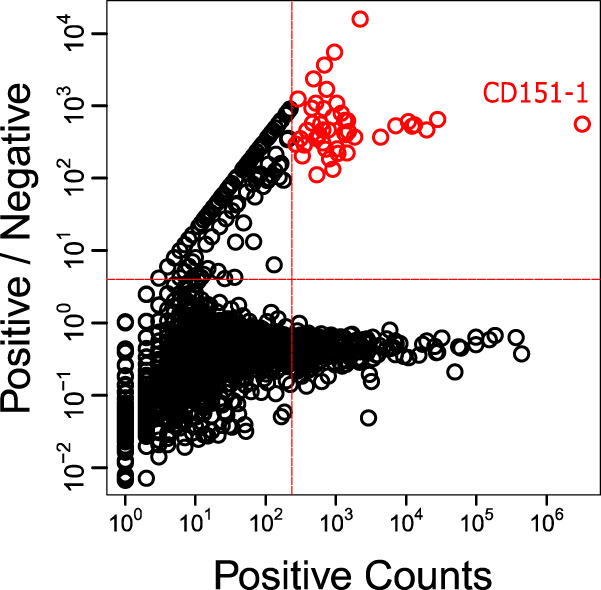
NGS enrichment ranking selection of CD151 in situ selection of Ab clones. The abundance of each sequence (N:105,434) in Fab-phage pools selected for binding to HEK293T-CD151+ cells (positive counts, x-axis) is plotted versus the ratio of the abundance in pools selected for binding to HEK293T-CD151+ cells over pools selected for binding to HEK293T-CD151− (positive/negative, y-axis). Each circle represents one unique paratope (i.e., unique combination of CDRs L3, H1, H2, and H3). The dashed red lines define an upper-right quadrant that contains putative CD151 binding clones, defined arbitrarily as those occurring more than 200 times in the positive pool and being greater than fourfold enriched relative to the negative pool. The red circles represent the 100 Ab clones in the upper-right quadrant that were selected after the NGS analysis and predicted to bind to CD151. All selected clones are close homologs (>80% sequence identity) of the immunodominant Ab clone CD151-1. The red circle at the far right represents the immunodominant Ab clone CD151-1 that was manually sampled and validated as an anti-CD151 Ab by cellular phage ELISA (Fig. S5).

To perform NGS Ab enrichment ranking selection of potential CD151 selective clones, the unique high-quality sequence reads from each pool were parsed based on CDR sequences and observation counts. For each unique sequence we plotted the counts in the positive pool (x-axis) versus the ratio of its abundance (i.e., frequency) in the positive pool relative to the negative pool (y-axis) (Fig. 3). To predict anti-CD151 specific Abs, we interrogated the sequences found in the upper-right quadrant corresponding to sequences with observations counts greater than 200 in the positive pool, and more than fourfold enrichment relative to the negative pool (Fig. 3).

The enrichment of unique sequences in the positive pool compared to the negative pool is highly variable (Fig. 3), spanning from highly enriched (y-axis >10^4^) to highly depleted (y-axis <10^−2^). Moreover, the sequences that are abundant in the positive pool show two distinct distributions (tails) based on their enrichment in comparison with the negative pool (Fig. 3): one tail is seen in the upper-right quadrant and corresponds to sequences that are abundant in the positive pool and much less abundant in the negative pool, while the second tail on the bottom-right quadrant contains sequences that are abundant in both positive and negative pools. The strategy of CellectSeq in situ selection against cell-surface CD151 on live cells resulted in high diversity (105,434 and 251,621 unique amino acid sequences in the positive and negative pools, respectively), expanding both specific and nonspecific clones (large number of clones in both upper-right and bottom-right quadrants), but are discriminable by comparing their abundances in the positive pool and the negative pool. After comparing all unique sequences, the NGS enrichment ranking revealed all upper-right quadrant clones as being close homologs of clone CD151-1, sharing >80% sequence identity in L3 and H3 sequences. This finding reveals that the Ab selection is enriched for homolog clones with a potentially similar targeted epitope (immunodominant), where CD151-1 is the most abundant and specific clone.

### Motif-based algorithm identifies selective and diversified Abs binding to CD151

**Fig. 4:**
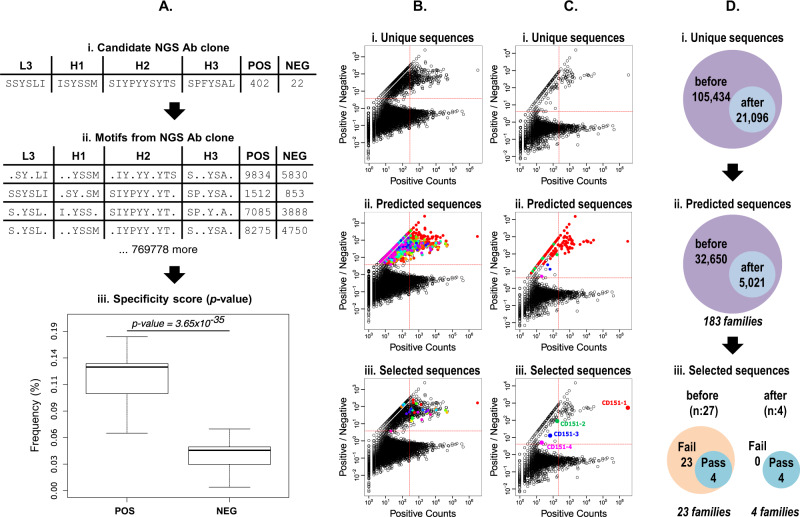
CellectSeq motif-based Ab discovery strategy of CD151 selective Abs. **A** Scoring Specificity: (i) Consider the candidate antibody NGS clone, the CDR sequences and counts in the positive (POS) and the negative (NEG) selection pools at round 4 are reported. (ii) We enumerate all consensus motifs in the CDR sequences. (iii) We score the binding specificity of the candidate antibody by assessing the separation between the distributions of the frequencies of the motifs in the positive (POS) and negative (NEG) pools. To this end, we calculate the *p* value of the *t*-test (Methods). **B**, **C** In each figure, the abundance of each sequence in the positive pool (positive Counts, x-axis) is plotted versus the ratio of the abundance in the positive pool over the negative pool (positive/negative, y-axis). Each circle represents one unique Ab clone, colored circles (except black) represent families of homologous sequences (sequence identity >0.75). The dashed red lines define an upper-right quadrant that contains enriched Ab clones defined as occurring more than 200 times in the positive pool and being greater than fourfold enriched relative to the negative pool. In contrast to Fig. 3, the dashed red lines here serve only as visual references to inspect the individual enrichment levels of the clones predicted by CellectSeq as highly specific (*p* value <10^−10^). **B** Before Filtering: NGS sequences and predicted sequences before filtering hybridization errors (N:368,427). (i) Distribution of unique sequences in the positive pool and their enrichment over the negative pool. (ii) Distribution of predicted sequences with high specificity (*p* value <10^−10^), colored by family of homologs. (iii) Distribution of selected sequences for in situ validation, colored by family of homologs. **C** After filtering: NGS sequences and predicted sequences after filtering hybridization errors (N:105,434). (i) Distribution of unique sequences in the positive pool and their enrichment over the negative pool. (ii) Distribution of predicted sequences with high specificity (*p* value <10^−10^), colored by family of homologs. (iii) Distribution of selected sequences for in situ validation, colored by family of homologs. Colored circles represent Ab clones named CD151-1 to CD151-4 which were selected and validated as specific CD151 binding Abs by cellular phage ELISA. Clones CD151-2 to CD151-4 are in the top-left quadrant, which means they are outside the scope of the NGS enrichment ranking method. **D** Before versus After filtering: Difference between number of unique sequences and prediction results before and after filtering hybridization errors. (i) Number of unique sequences in the positive pool before and after filtering. (ii) Number of predicted sequences before and after filtering. (iii) Number of validated sequences before and after the filtering.

Due to the lack of Ab diversity derived either from manual selection of Ab clones or NGS enrichment ranking, we developed a motif-based algorithm to identify highly selective Abs for CD151 from the deep sequenced phage pools. The in silico strategy for scoring CD151 selective Abs is based on exploring all possible sequence motifs (i.e., consensus motifs) in the positive pool and scoring their enrichment over the negative pool (Fig. 4A). This follows the premise that highly selective Abs are enriched with paratope motifs (i.e., linear information) that recognize the target antigen, whereas non-selective Abs lack such enrichment^49^ (Fig. S7). Therefore, for each Ab clone in the positive pool (i.e., candidate) (Fig. 4Ai) we explored the entire space of linear information by exhaustively enumerating all possible motifs matching its CDR sequences^50^, and obtained the frequencies (number of matching sequences/total number of sequences) of every motif in the positive and negative pools (Fig. 4Aii). According to the premise above, the high enrichment of the motifs in the positive pool relative to the negative pool implies the Ab candidate is potentially highly selective (Fig. 4Aiii). Thus, we analyzed each Ab in the positive pool for the selective binding to CD151 by scoring the separation between the two distributions of frequencies of the motifs in the positive and negative pools (see Methods for details). To this end, we calculated the *t*-test^51^ to score the separation of the two distributions, then we calculated the *p* value^52–54^ to evaluate the statistical significance of the *t*-test (Fig. 4Aiii). Thus, the lower the *p* value, the higher the separation between the two distributions, and consequently, the higher the selectivity of the candidate Ab. Finally, we applied the stringent *p* value cut-off of 10^−10^ to identify highly selective Ab clones (see Methods). Therefore, this motif-based in silico strategy allowed us to explore rapidly and exhaustively the selectivity of all Ab clones in the positive pool. We were able to identify potential selective CD151 binders, regardless of their individual frequencies in the total pool of sequences, thus bypassing the limitations of standard NGS analyses based solely on enrichment counts of individual clone sequences^55–62^, which make them inapt for identifying selective Ab clones with low frequencies.

### Filtering PCR-induced sequence artifacts improves the in silico Ab selection results

As previously mentioned, PCR-induced artifacts may arise during the NGS sample preparation and Illumina sequencing process^45^. These artifacts represent invalid amalgams of existing CDRs H1, H2, H3, and L3 sequences, which may be seen as novel Ab clones^45^. These artifacts may significantly bias the frequencies of individual clones that will inevitably affect the in silico Ab discovery strategy. Therefore, for both the positive and negative pools, we obtained the frequencies (i.e., number of observations) of L3/H3 pairs, where both CDRs are the most diverse in terms of length and amino acid compositions in Library F^42^. We calculated a frequency cut-off to determine valid L3/H3 pairs utilizing a minimal occurrence threshold, with all invalid pairs filtered from the selection pool as potential PCR and NGS artifacts (see Methods). We then applied the motif-based in silico Ab discovery strategy to predict CD151 highly selective binders (*p* values <10^−10^) for both scenarios, before and after filtering (Fig. 4Bii, Cii). The error-filtering eliminated 80% of the unique clones from the positive pool (Fig. 4Bi, Ci, Di). Similarly, the application of the error-filtering before the motif-based in silico prediction of CD151 clones eliminated 85% of the unique Abs (Fig. 4Bii, Cii, Dii). Interestingly, before error-filtering the in silico predicted Abs clustered into 183 distinct families of similar L3/H3 sequences (>80% identity), whereas after filtering the Abs reduced to only four distinct families (Fig. 4Biii, Ciii, Diii).

To experimentally assess the validity of predicted Abs in both scenarios, we selected the Abs with best specificity scores (*p* values; Fig. 4Aiii) from each of the four families predicted after filtering, as well as 23 additional Abs predicted before filtering (Fig. 4Biii, Ciii, Diii and Table S1). Due to the low NGS enrichment of the identified Ab clones, instead of PCR rescue or similar methods^63,64^, genes encoding the 27 candidate Ab clones were synthesized and cloned into expression plasmids to permit recombinant production of Fab protein from *E. coli*. After purification, we compared the activity of each Fab by flow cytometry for binding to HEK293T-CD151+ cells relative to HEK293T-CD151− cells. All four Ab clones predicted after filtering were validated experimentally as CD151 binders (Pass validation; Table S1). On the other hand, all 23 pre-filtering Abs failed to bind to CD151-expressing cells (Table S1). Thus, the success rate of the motif-based in silico Ab discovery before and after filtering was 15% or 100%, respectively. This difference between the success rates highlights the requirement to filter PCR-induced and NGS artifacts to reveal bona fide selective clones. Furthermore, the abundances of the four identified clones (based on motif-based in silico Ab selection) varied from high (30%) to extremely low. In fact, the clones CD151-2 and CD151-3 had frequencies below 0.001%, and the frequency of clone CD151-4 was extremely low (0.00000093%, Table 1).

### Characterization of motif-based in silico identified anti-CD151 Abs

**Fig. 5:**
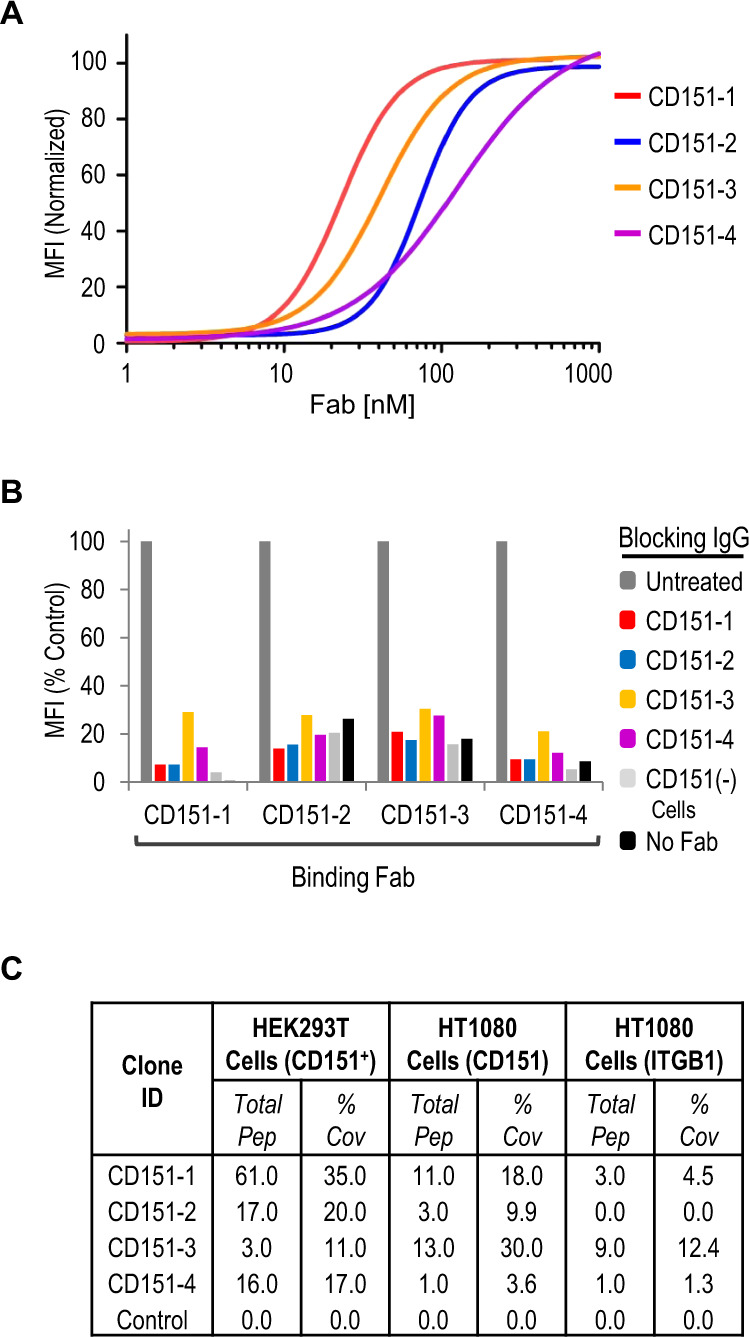
Characterization of anti-CD151 Abs derived from CellectSeq. **A** Dose response curves for anti-CD151 Fabs assessed by flow cytometry fluorescence (y-axis) using HEK293T-CD151+ cells. The MFI signals were subtracted from background Fab binding to HEK293T-CD151− cells and normalized to the highest concentration value for each sample. Data a representative of *n* = 2 biologically independent experiments. **B** Blocking of anti-CD151 Fabs (x-axis) binding to HEK293T-CD151+ cells by indicated IgGs, assessed by flow cytometry fluorescence (y-axis). Data a representative of *n* = 2 biologically independent experiments. **C** Mass-spectrometry summary table of enriched isolated peptides from immuno-precipitated CD151 cellular lysates from HEK293T-CD151+ or HT1080 cells with anti-CD151 Fabs or control Fab. Percent coverage is the percentage of CD151 protein detected by the total peptides.

To demonstrate the advantage of the motif-based in silico Ab discovery strategy, termed CellectSeq (Fig. 1), we measured all four Fab (CD151-1 thru -4) for dose-dependent binding to HEK293T-CD151+ cells. Quantitative flow cytometry displayed tight and saturable binding (Fig. 5A), with EC50 values in the low nanomolar range (Table 1). We also used flow cytometry to assess epitope overlap by measuring the ability of immunoglobulin (IgG) versions of each clone to block binding of each Fab to HEK293T-CD151+ cells. As expected, preincubation of HEK293T-CD151+ cells with each IgG reduced subsequent binding of the cognate Fab. Moreover, all IgGs blocked binding of the other Fabs (Fig. 5B), implying that all four Fabs share overlapping epitopes.

Further corroboration of specificity for CD151 was provided by performing IP-MS experiments with each Fab with HEK293T-CD151+ cells and HT1080 cells that express endogenous CD151. Tandem mass spectra were searched against a human database to validate MS/MS protein identifications. Protein identifications were accepted if they could be established at greater than 99% probability based on identified peptides. After background filtering to remove keratin, IgG and cytoplasmic proteins, the highest peptide counts for all four Fabs were for CD151 on both cell lines (Figs. 5C and S8). The integrin β1 (ITGB1), a receptor identified to associate with CD151^65^, also immunoprecipitated with Fabs CD151-1, CD151-3, and CD151-4 (Fig. 5C). Taken together, the results show that the four Abs recognize cell-surface CD151 with high affinity and specificity.

### CellectSeq identifies diverse and selective Abs binding to CA9 and integrin-α11

To further validate CellectSeq, we expanded our analysis with two additional transmembrane proteins. We targeted CA9, a cell-surface enzyme associated with remodeling the tumor pH environment^38,39^, and integrin-α11β1, a cell-surface heterodimeric receptor involved in cancer-associated fibroblast biology^36,40,41^. We performed independent in situ selections against cell-surface CA9 and integrin-α11 on live cells displaying each antigen. To enrich binders for CA9, we engineered HEK293T cells to stably overexpress CA9 (HEK293T-CA9+, positive cells), and to deplete non-target selective binders we utilized the parental HEK293T cells (HEK293T-WT, negative cells). To enrich binders for integrin-α11, we engineered C2C12 cells to stably overexpress integrin-α11 (C2C12-α11+, positive cells), and to deplete non-selective binders we utilized the parental C2C12 cells (C2C12-WT, negative cells). We then performed the same in situ selection strategy as for CD151 (Fig. 2), composed of four rounds of selection against positive and negative cells to produce a positive Ab pool enriched with selective clones for the target antigen and a negative Ab pool enriched with non-specific clones.

Following selections, we performed NGS on positive and negative pools for each target. We followed the same NGS procedure as described for CD151 to obtain positive and negative pools of sequences for each target. For CA9 we obtained 1,793,961 (Supplementary Data 3) and 1,061,005 (Supplementary Data 4) high quality NGS reads for the positive and negative pools, respectively. Translation of the reads yielded 257,692 and 73,380 unique amino acid sequences in the positive and negative pools, respectively. For integrin-α11 we obtained 4,440,015 and 1,750,677 reads for the positive and negative pools, respectively, and translation yielded 108,441 (Supplementary Data 5) and 47,674 (Supplementary Data 6) unique amino acid sequences in the positive and negative pools, respectively.

**Fig. 6:**
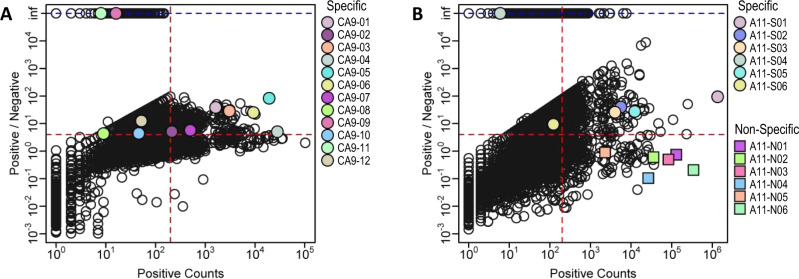
Predicted unique Ab clones by CellectSeq on CA9 and integrin-α11 NGS data. **A**, **B** Each circle represents a unique Ab clone (i.e., a unique combination of CDRs L3, H1, H2, and H3) generated by NGS. The abundance of each sequence in the Fab-phage pool selected for binding to target antigen overexpressing cells (x-axis: positive counts) is plotted versus the ratio of the abundance in the pool selected for binding to antigen overexpressing cells over pools selected for binding to parental cells (y-axis: positive/negative). The dashed blue line (y-axis: inf) corresponds to the clones with zero observations in the negative pool. The dashed red lines define an upper-right quadrant that contains putative binding clones, defined arbitrarily as those occurring more than 200 times in the positive pool and being greater than fourfold enriched relative to the negative pool. In contrast to Fig. 3, the dashed red lines here serve only as visual references to inspect the individual enrichment level of the clones predicted by CellectSeq as highly specific (*p* value < 10^−10^). **A** Predicted unique Ab clones by CellectSeq on CA9 NGS data. Colored solid circles (N:257,692) represent the twelve clones from distinct families of L3/H3 sequences (>80% sequence identity in L3 and H3) predicted with the highest specificity (lowest *p* value <10^−10^). Specificity for CA9 was predicted by CellectSeq and validated by cellular phage ELISA. **B** Predicted unique Ab clones by CellectSeq on integrin-α11 NGS data. Colored solid circles (N:108,441) represent the six non-homolog clones (>80% sequence identity in L3 and H3) predicted with the highest specificity (lowest *p* value <10^−10^). Colored solid squares represent the six most enriched clones from distinct families of L3/H3 sequences (>80% sequence identity in L3 and H3) in the positive pool predicted as nonspecific (highest *p* value >10^−3^). Specificity for integrin-α11 was predicted by CellectSeq and validated by cellular phage ELISA.

Applying the same methods described above for CD151, we selected twelve candidate Abs for CA9 (CA9-01 to CA9-12) (Fig. 6A and Table 1). By ELISA, all 12 purified Fabs bound selectively to HEKT293-CA9+ cells compared with HEKT293T-WT cells (Table 1 and Fig. S9). Many clones exhibited extremely low abundances (e.g., CA9-08, CA9-09, and CA9-11 frequency below 0.00001%), and these would have required extensive screening to discover by conventional methods.

For integrin-α11 we selected six candidates (A11-S01 to A11-S06) (Fig. 6B and Table 1) and all six Fabs bound selectively to C2C12-α11+ cells compared with C2C12-WT cells (Table 1 and Fig. S9). To further validate CellectSeq, we also applied the motif-based in silico Ab discovery strategy to predict non-selective Ab binders from the integrin-α11 data. We selected six unique and diverse Abs with high abundances, yet with poor specificity scores (*p* values >10^−3^), from distinct families of L3/H3 sequences (A11-N01 to A11-N06) (Fig. 6B and Table 1). As predicted, none of the six Fab proteins bound integrin-α11 selectively (Fig. S9). Taken together, CellectSeq was successful in using NGS data for positive and negative pools to identify selective but extremely low frequency Abs for three distinct classes of membrane proteins.

## Discussion

Membrane proteins represent about 70% of current drug targets^14^. However, the limited cell-surface component of many integral membrane proteins makes their production and purification extremely difficult for in vitro Ab selections^13^. The instability of membrane proteins makes them challenging targets to work with, as many of these proteins depend on the membrane environment for their correct structure and function. The Ab selection strategy presented in this work, termed CellectSeq (Fig. 1), bypasses the need for purified antigens by performing selections directly on cell-surface antigens. Moreover, CellectSeq may target difficult receptors, such as those containing minimal loop protrusions and those present in complex mixtures in situ, as is the case for tetraspanin receptors.

The in situ Ab selection against CD151 followed by conventional manual screening yielded a unique immunodominant clone. NGS enrichment ranking analysis of the same selection identified highly homologous clones, with greater than 80% sequence identity in CDRs L3 and H3. In contrast, CellectSeq combined sequencing error-filtering and motif-based scoring algorithms to identify candidate Ab clones in NGS data, whether enriched or depleted, but with high statistical scores for specificity. As a result, CellectSeq surpasses conventional strategies for applying NGS to derive Abs, such as enrichment ranking^55–62,66^, by performing an exhaustive analysis of all paratope motifs in the NGS dataset, rather than unique observations of clonal sequence identities. This statistical evaluation of paralogs allows for the successful prediction of representative Abs against CD151, including low abundance clones at frequencies as few as one in a million reads. Nevertheless, all four distinct paratopes identified by CellectSeq share a similar target epitope (Fig. 5B), highlighting the recalcitrant nature of CD151^35^, which displays limited surface epitopes (Figure S1).

We also credited the success of CellectSeq to the design of the synthetic Ab repertoire itself. In contrast to existing strategies for enhancing sequencing fidelity in Illumina datasets^67–70^, the simple and defined design of Library F permits simple and accurate detection of erroneous Illumina reads. Library F was constructed on a single human fixed framework with diversity restricted to only four CDRs with defined length and chemical compositions. This library allows for efficient NGS data acquisition based on pair-end reads with three primers. Furthermore, the design of positional codon frequencies in the restricted CDRs allows for rapid deconvolution of NGS datasets and the removal of errors and artifacts, whereas natural repertoires are more diverse and undefined. Additionally, the synthetic CDRs enable a deep analysis of paratope diversities in the NGS data by utilizing motif-based in silico strategies that predict infrequent but target-specific Abs, as demonstrated by the successful prediction of rare but selective Abs for CD151, CA9, and integrin-α11.

In recent years, a variety of methods using NGS for antibody discovery have been developed. Most of the methods exclusively explore CDR-H3 repertoires^55,57–59,61,62^. The identified potential binders in NGS data are discovered using naïve approaches, including enrichment ranking or interrogation of antibody databases^55–62^. Accordingly, the exclusive exploration of CDR-H3 is mainly inspired by natural Ab repertories, in which CDR-H3 tends to drive most of the affinity and specificity, primarily due to the longer CDR loop allowing for increased structural diversity. In contrast, synthetic Ab libraries are based on defined CDR amino acid composition and length parameters.

The synthetic library F was constructed with equivalent chemical diversity in CDRs H3 and L3. This feature contrasts with natural Abs in which CDR-H3 drives affinity capture and is more diverse than CDR-L3 due to the genetic mechanisms that generate antibody encoding genes. Therefore, the primary drivers of antigen affinity in Library F are both CDR-L3 and CDR-H3. Interestingly, we previously showed that CDR-L3 in Library F is sufficient to drive antigen affinity without assistance from CDR-H3^42^. This implies that CDR-H3 is not necessarily required for generation of Abs that recognize diverse protein antigens, and CDR-L3 may assume the dominant role for antigen recognition. It also implies that the combination of CDR-L3 and CDR-H3 offers higher diversities than CDR-H3 alone (Fig. S2D), with increased numbers of potential binders when compared to the methods that explore CDR-H3 repertoires exclusively^55,57–59,61,62^.

NGS data commonly include many potential errors^45,62,71^. For instance, PCR is a major contributor to sequencing errors, since it induces biases and artifacts that inevitably arise during sample preparation and sequencing. As a result, these errors skew the distribution of PCR products due to unequal amplification of clones and DNA polymerase efficiency. Therefore, NGS procedures have been greatly optimized to minimize sequencing errors, but they inevitably still contain errors^45,62,71^. For this reason, CellectSeq includes post-sequencing steps for filtering sequencing errors and PCR artifacts. As demonstrated (Fig. 4), the incorporation of error-filtering in NGS analysis is essential for the identification of rare selective clones by applying the motif-based algorithm, for which the methods based on enrichment ranking in NGS data are inadequate because rare clones are not distinguishable among background and errors.

In situ selections against native antigens on live cells within the heterogeneous cell surface protein mixture would inevitably enrich both selective and nonselective Abs. However, selections against positive and negative cells produce pools enriched with selective and nonspecific clones, respectively. NGS enrichment analysis is then able to discern target specific clones in the positive pool by comparison with the negative pool^58,60,62,72^. However, we show that NGS enrichment ranking alone proves inadequate for identifying rare but selective clones that are not readily distinguishable from background. By applying CellectSeq, which includes a post-sequencing in silico analysis for filtering errors and scoring specificity, we were able to identify unique target selective clones independent of frequency counts. CellectSeq therefore enhances the output clonal diversities of potential Ab candidates and increases the pool of potential functional Abs.

The implementation of NGS-based in silico analysis facilitates the rapid and successful discovery of Abs, and highlights that membrane associated antigens are accessible to synthetic Abs in situ. As demonstrated in this report, the strategy of CellectSeq surpasses standard methods of Ab manual screenings and NGS analysis to interrogate all potential binders in the output Ab pool. Additionally, because NGS is an attractive technology for generating meta-data, regarding costs and access for Ab discovery^73,74^, we foresee the expanded implementation of CellectSeq to help identify diversified paratopes and low abundance clones. This is evidenced by recent reports of deep sequencing approaches that characterize human Ab libraries and V-gene repertoires from immunized mice^75,76^. Therefore, by combining synthetic Ab libraries, in situ selections, NGS, and in silico analysis, CellectSeq may open the door for proteomic scale generation of Abs.

## Methods

### Cell lines and culture practices

Both, the CD151 knockdown (HEK293T-CD151−) and CD151 overexpressing (HEK293T-CD151+) cell lines were gifts from the Dr. Rottapel lab at University of Toronto, Princess Margaret Cancer Centre. Briefly, the HEK293T-CD151− cells were generated using the Tet-pLKO-puro plasmid and the HEK293T-CD151+ cells were generated using the pLX304 plasmid, both as previously described^77^. The overexpressing integrin-α11 (C2C12-α11+) cells were previously described^36^, and the CA9 overexpressing (HEKT-CA9+) cells were a gift from Northern Biologics Inc. and generated as previously described^78^, which stably introduced *CA9* ORF cDNA (OriGene Technologies) into the cell line genome. The HEK293T and C2C12 cell backgrounds were cultured in Dulbecco’s Modified Eagle medium (DMEM) with 10% fetal bovine serum (FBS). The human fibrosarcoma H1080 cell line (ATCC; CCL-121) was cultured in Eagle’s Minimum Essential Medium (EMEM) with 10% FBS. All cells were cultured at 37 °C in a humid incubator with 5% CO2.

### Design of synthetic antibody Library F

The synthetic Library F was previously described^42^. It was constructed by enabling the incorporation of desirable design features to enhance antibody performance and lower the risk of immunogenicity for therapeutic applications. Library F was built on a single framework with diversity restricted to only four CDRs (L3, H1, H2, and H3), and at positions most likely to enhance function without compromising structure. A restricted diversity of primarily tyrosine and serine was added to CDRs H1 and H2 because natural antibody antigen-binding sites are enriched for tyrosine and serine^79^. Both these amino acid residues are intrinsically well suited for molecular recognition, where tyrosine sidechains establish binding contacts with the antigen and the small serine sidechains serve as auxiliaries that help to position Tyr in favorable binding conformations^80^. Also, amino acid and CDR length diversities were added to CDR-L3 and CDR-H3 using only nine distinct amino acids (Fig. S2) these were selected for their relative abundance bias at protein–protein interfaces and binding hotspots^81,82^. Additionally, amino acid diversity was added to non-paratope residues to further augment CDR conformations. Accordingly, such minimalistic synthetic antibody design permits sufficient conformation and chemical diversity to mediate high-affinity recognition of target antigens^42,82^.

### Antibody selections with cellular antigen

For CD151, phage pools representing synthetic antibody library-F^42^ were cycled through four rounds of binding selections using a HEK293T-CD151− cell line as the background depleting step, and a HEK293T-CD151+ cell line as the target selection step (Fig. 2). The adherent cell lines were suspended using PBS, 10 mM ethylenediaminetetraacetic acid (EDTA) (Sigma-Aldrich). For round 1, ten million re-suspended HEK293T-CD151+ cells (greater than 90% viability) were incubated with Fab-phage (3 x 10^12^ cfu) in cell growth media under gentle rotation for 2 h at 4 °C. For rounds 2 and 3, the Fab-phage were cycled between antigen negative (to remove non-specific phage binders) to antigen positive cells. Here the Fab-phage were first incubated with the HEK293T-CD151− cell line for 2 h at 4 °C, then the cells were spun down utilizing a chilled centrifuge and Fab-phage supernatant collected. Similarly, the HEK293T-CD151+ cells were spun down utilizing a chilled centrifuge and supernatant discarded. Next, the HEK293T-CD151+ cells were resuspended utilizing the Fab-phage supernatant, and incubated for 2 h at 4 °C. For round 4, both HEK293T-CD151− and HEK293T-CD151+ cells were independently presented with Fab-phage and incubated for 2 h at 4 °C. The HEK293T-CD151− and HEK293T-CD151+, cell lines were washed four times with chilled PBS and 1% BSA.

For all rounds, after washing the bound phages were eluted from the cell pellet by resuspending the cells in 0.1 M hydrochloric acid and incubating for 10 min at room temperature. The cell solutions were neutralized using 11 M Tris buffer (Sigma-Aldrich), cellular debris was removed by high-speed centrifugation, and the eluent was transferred to clean vials. The output phages were amplified by infection and growth in *E. coli* OmniMAX^TM^ cells (Thermo-Fisher). After round 4, infected *E. coli* OmniMAX^TM^ cells were plated on 2YT/carbenicillin (Sigma-Aldrich) plates for isolation of single colonies. For CA9 and integrin-α11 we performed the same in situ selection strategy as for CD151 (Fig. 2). To enrich binders for CA9, we utilized HEK293T cells that stably overexpress CA9 (HEK293T-CA9+; positive cells), and to deplete non-target selective binders we utilized the parental HEK293T cells (HEK293T-WT; negative cells). To enrich binders for integrin-α11, we utilized C2C12 cells that stably overexpress integrin-α11 (C2C12-α11+; positive cells), and to deplete non-target selective binders we utilized the parental C2C12 cells (C2C12-WT; negative cells).

### ELISAs

Colonies of *E. coli* OmniMAX harboring phagemids were inoculated into 450 µl 2YT broth supplemented with carbenicillin and M13-KO7 helper phage, and the cultures were grown overnight at 37 °C in a 96-well format. Culture supernatants containing Fab-phage were diluted twofold in PBS buffer supplemented with 1% BSA and incubated for 15 min at room temperature. To test binding to native antigen on cells, phages were added directly to the cellular media of HEK293T-CD151− and HEK293T-CD151+adherent cells (95–100% confluence) in tissue-culture-treated 96-well plates (Thermo-Fisher). After incubation for 45 min at room temperature, the plates were washed gently with PBS and the cells were fixed with 4% paraformaldehyde (Sigma-Aldrich). The cells were washed with PBS and incubated for 30 min with horseradish peroxidase/anti-M13 Ab conjugate (Sigma-Aldrich) in PBS buffer supplemented with 1% BSA. The plates were washed, developed with TMB Microwell Peroxidase Substrate Kit (KPL Inc.), and quenched with 1.0 M phosphoric acid; the absorbance was determined at a wavelength of 450 nm. Clones were identified as positive if they produced at least fivefold greater signal on wells with HEK293T-CD151+cells over antigen negative HEK293T-CD151− cells. All positive clones were subjected to Sanger DNA sequence analysis (Genewiz). For Fab cellular ELISAs against CA9 and integrin-α11 a final 250 nM Fab concentration was added to the cellular media of antigen overexpressing or parental adherent cells (95–100% confluence) in tissue-culture-treated 96-well plates (Thermo-Fisher). After incubation for 45 min at room temperature, the same procedure previously described as for CD151 was followed.

### Fab protein purification

Fab proteins were expressed in *E. coli* BL21 (ThermoFisher), as described^43^. Following expression, cells were harvested by centrifugation and cell pellets were flash-frozen using liquid nitrogen. The cell pellets were thawed, re-suspended in lysis buffer (50 mM Tris, 150 mM NaCl, 1%Triton X-100, 1 mg/ml lysozyme, 2 mM MgCl_2_, 10 units of benzonase), and incubated for 1 h at 4 °C. The lysates were cleared by centrifugation, applied to rProtein A-Sepharose columns (GE Healthcare), and washed with 10 column volumes of 50 mM Tris, 150 mM NaCl, and pH 7.4. Fab protein was eluted with 100 mM phosphoric acid buffer, pH 2.5 (50 mM NaH_2_PO_4_, 140 mM NaCl, 100 mM H_3_PO_4_) into a neutralizing buffer (1 M Tris, pH 8.0). The eluted Fab protein was buffer exchanged into PBS and concentrated using an Amicon-Ultra centrifugal filter unit (EMD Millipore). Fab protein was characterized for purity by SDS-PAGE gel chromatography and concentration was determined by spectrophotometry at an absorbance wavelength of 280 nm.

### IgG purification

Full-length IgG proteins were expressed in mammalian cells, as described^83^. Briefly, plasmids designed to express heavy and light chains were co-transfected into Expi293 cells (ThermoFisher) using the FuGENE^®^ 6 Transfection Reagent kit (Promega), according to the manufacturer’s instructions. After 5 days, cell culture media was harvested and applied to an rProtein-A affinity column (GE Healthcare). IgG protein was eluted with 25 mM H_3_PO_4_, pH 2.8, 100 mM NaCl and neutralized with 0.5 M Na_3_PO_4_, pH 8. Fractions containing eluted IgG protein were combined, concentrated, and dialyzed into PBS, pH 7.4. IgG protein was characterized for purity by SDS-PAGE gel chromatography and concentration was determined by spectrophotometry at an absorbance wavelength of 280 nm.

### Flow-cytometry validations and cellular binding titrations

Adherent cells were brought into suspension using PBS supplemented with 10 mM ethylenediaminetetraacetic acid (EDTA; Sigma-Aldrich). The cells were washed with PBS, resuspended in PBS supplemented with 1% BSA, and incubated for 15 min at 4 °C. The cells were labeled with 500 nM Fab, or IgG for 30 min at 4 °C, then washed with PBS and resuspended in PBS supplemented with 1% BSA. Next, the cells were labeled with anti-Flag (for Fabs) conjugated Alexa-488 secondary Ab (Abcam) according to the manufacturer’s instructions. Data were collected using a CytoFLEX-S flow-cytometer (Beckman Colter) using a 488-nm laser with 530/25 nm filter settings. The cells were analyzed in PBS, and all acquired live events (Fig. S10) were greater than 10,000 cells per sample. Quantitation analysis was carried out using FlowJo v10.2 Software (FlowJo, LLC). For Ab cellular titration analysis, the Abs were added to antigen positive HEK293T-CD151+ cells in triplicate samples from a concentration range of 500 pM to 1 µM. The mean fluorescence signal values were subtracted from the control antigen negative HEK293T-CD151− cells signals (Fig. S11), and EC_50_ determined using Graph-pad Prism (GraphPad Software, San Diego, California, USA), where x is the Fab concentration:

1 $$Y={Y}_{{\mathrm{max}}}+\frac{{Y}_{{\mathrm{min}}}-{Y}_{{\mathrm{max}}}}{1+{\big(\frac{{\mathrm{EC}}50}{X}\big)}^{{\mathrm{HillSlope}}}}$$

### Mass Spectrometry

For immunoprecipitation of cell-surface protein, 10^7^ lifted and dissociated HEK293T-CD151+ or HT1080 cells were incubated with 500 nM Fab protein in DPBS with calcium and magnesium (Gibco, 0404) for 1 h at 4 °C. Cells were washed with PBS and lysed using IP lysis buffer (50 mM Tris-HCl pH 7.5, 150 mM NaCl, 1.0% IGEPAL CA-630, 0.25% Na-deoxycholate, 1 mM EDTA, and protease inhibitor cocktail (Roche)) for 15 min at 4 °C and centrifuged at 12,000 × *g* for 5 min at 4 °C. The supernatant was incubated with 30 μl of sepharose protein-A beads (GE Healthcare) for 1 h at 4 °C. The beads were washed three times with lysis buffer, once with PBS, and resuspended in 22 μl 10 mM glycine, pH 1.5. After 5 min, the supernatant was collected and neutralized with 2.2 μl 1 M Tris, pH 8.8. DTT was added to a final concentration of 10 mM. The sample was incubated at 40 °C for 1 h and cooled to room temperature. Iodoacetamide was added to a final concentration of 20 mM, and the sample was incubated at room temperature in the dark for 30 min. Trypsin (1 µg, Promega) was added, and the sample was incubated overnight at 37 °C. Peptides were purified using C18 tips and analyzed on a linear ion trap-Orbitrap hybrid analyzer (LTQ-Orbitrap, ThermoFisher) outfitted with a nanospray source and EASY-nLC split-free nano-LC system (ThermoFisher).

Tandem mass spectra were extracted, and charge state was deconvoluted and deisotoped by Xcalibur version 2.2. All MS/MS samples were analyzed using PEAKS Studio (Bioinformatics Solutions, Waterloo, ON Canada; version 8.0 (2016-09-08)) and X! Tandem (The GPM, thegpm.org; version CYCLONE (2010.12.01.1)). Samples were searched against the Uniprot Human database (Downloaded May 1, 2017: 20,183 entries) assuming the digestion enzyme trypsin. Carbamidomethyl of cysteine was specified as a fixed modification. Deamidation of asparagine and glutamine were specified as variable modifications. Scaffold (version Scaffold_4.7.5, Proteome Software Inc., Portland, OR) was used to validate MS/MS based peptide and protein identifications. Peptide identifications were accepted if they could be established at greater than 95% probability. Peptide Probabilities from PEAKS Studio (Bioinformatic Solutions, Inc.) were assigned by the Peptide Prophet algorithm with Scaffold delta-mass correction. Peptide Probabilities from X! Tandem were assigned by the Scaffold Local FDR algorithm. Protein identifications were accepted if they could be established at greater than 99% probability and contained at least one identified peptide. Protein probabilities were assigned by the Protein Prophet algorithm^42^. Proteins that contained similar peptides and could not be differentiated based on MS/MS analysis alone were grouped to satisfy the principles of parsimony.

### Next-generation sequencing analysis

The Fab-phage output pools from HEK293T-CD151− and HEK293T-CD151+ cell lines were utilized as input templates of PCR reactions using forward and reverse primers that flanked CDRs L3 and H3, respectively. The primers included a 24 bp template annealing region followed by a 6–8 bp unique nucleotide barcode identifier and an Illumina universal adapter tag (PE1 or PE2 for the reverse or forward primer, respectively). Duplicate PCR amplicons were generated per Fab-phage pool that were then isolated by gel electrophoresis and followed by agarose gel extraction (Qiagen). The duplicate PCR amplicons were combined, and the sample concentrations were determined by spectrophotometry (BioteK). The amplicons for antigen positive and negative Fab-phage pools were normalized, pooled, and sequenced using a HiSeq 2500 instrument (Illumina) with 300 paired-end cycles. Besides PE1 and PE2 Illumina universal primers, the sequencing runs also included a custom primer that allowed for complete sequencing of CDRs H1 and H2. Thus, the three primer reads together provided complete sequence coverage of the four CDRs that were diversified in Library F^42^ (Figs. S2 and S3). The NGS runs were performed using two distinct samples taken from the same parent sample resulting in duplicate NGS runs with high correlation and high overlap for unique sequences. The NGS output data from both independent runs were then merged to maximize the frequency and the number of unique sequences. Subsequently, the sequencing reads were deconvoluted for each clone, and the three primer reads (PE1, PE2, and custom) were combined into a single sequence to derive the complete sequence. Sequences were filtered from sequencing errors using per base high quality score cut-off of *Q* = 30, which corresponds to 1:1000 of incorrect base call^43^. High quality nucleotide sequences were obtained, translated into amino acid sequences, and compared to the designed sequence repertoire of Library F^42^ to filter out technical errors inherent to sequencing and PCR amplification.

### Filtering hybridization errors in NGS selection pools

The diversity, i.e., combinatorial possibilities, of our phage-displayed synthetic Ab library^42^ is dominated by the CDR sequences L3 and H3 (Fig. S2D). In fact, the theoretical diversity of L3 and H3 are to the order of magnitude of 9 and 16, while H1 and H2 cover a diversity of 2^6^ and 2^8^. Thus, we theoretically assumed that most PCR-induced artifacts and bias representing amalgams of existing sequences, i.e., hybridization errors, to be present in the pairs of sequences L3/H3. In addition, we assumed that among all possible pairs of sequences L3/H3, the valid pairs are overrepresented compared to the invalid pairs. Thus, for every sequence H3 in the Ab selection pool, we obtained the frequencies (i.e., number of observations) of all its paired sequences L3, and we calculated a frequency cut-off according to the maximum interclass inertia method using the Koenig–Huygens theorem^84^. The cut-off serves as a minimum frequency threshold to identify valid pairs L3/H3, thus, all Ab sequences with the invalid pairs are filtered from the selection pool.

### Enumeration of consensus motifs in the CDR sequences

Consensus motifs, or motifs, are utilized to represent the linear information that is shared among groups of sequences. While certain positions in the motifs are defined (e.g., P as proline and R as arginine in the motif PXXR), others do not and are called wildcards (e.g., X as any amino acid in the motif “PXXR”). We utilize here the motifs to explore the linear information in the CDR sequences of each candidate Ab. To this end, we adapted the algorithm DALEL^50^ that was first developed to explore the linear information in proteins. To avoid the explosion of the number of motifs, we restricted the number of allowed wildcards in each motif to 55% of its length. In addition, we considered only motifs with wildcards matching more than one amino acid in the matching sequences in the positive pool (e.g., wildcard X in motif PXR matches amino acids Y and S in the sequences PYR and PSR). Finally, we restricted the number of motifs by limiting the minimum number of sequences in the positive pool matching every motif to a 100.

### Scoring separation between distributions of frequencies in positive and negative selections

The following procedure is performed for every Ab from the positive pool. We enumerate all possible motifs in the CDR sequences, as described earlier. Then, for each motif we obtain the frequencies (i.e., number of matching sequences/total number of sequences) in both the positive and the negative pools. According to the premise that selective Abs are enriched with paratope motifs that recognize the target antigen and non-selective Abs are not, it is then possible to assess Ab selectivity by checking whether the frequencies in the positive pool are higher compared to the negative pool. This is performed by scoring the level of separation between the distribution of frequencies in the positive and the negative pools.

To score the separation between the two distributions of frequencies of the motifs in the positive and the negative selection pools we utilized the Welch–Satterthwaite version of the *t*-test in conjunction with the rank transformation^51^. This approach has the advantage to simultaneously counteract the undesirable effects of both non-normality and unequal variances in our distributions of frequencies. First, the two distributions of frequencies are combined and arranged in ascending order, with tied frequencies receiving a rank equal to the average of their positions. Given the two distributions of ranks *x*_p_ and *x*_N_ that correspond to the frequencies in the positive and the negative selection pools, and ${\bar{x}}_{P}$ and ${\bar{x}}_{N}$ are the means, *s*_p_ and *x*_N_ are the standard deviations, and *n*_p_ and *n*_p_ are the sizes, all, respectively. We calculate the *p* value to evaluate the statistical significance of the *t*-score following the procedure described below^52–54^.

We first calculate the *t*-score by:

2 $$t=\frac{{\bar{x}}_{{\mathrm{p}}}-{\bar{x}}_{{\mathrm{N}}}}{\sqrt{\frac{{s}_{{\mathrm{P}}}^{2}}{{n}_{{\mathrm{P}}}}+\frac{{s}_{{\mathrm{N}}}^{2}}{{n}_{{\mathrm{N}}}}}}$$

We then calculate the degree of freedom by:

3 $$f=\frac{{\big(\frac{{s}_{{\mathrm{P}}}^{2}}{{n}_{{\mathrm{P}}}}+\frac{{s}_{{\mathrm{N}}}^{2}}{{n}_{{\mathrm{N}}}}\big)}^{2}}{\frac{{\big(\frac{{s}_{{\mathrm{P}}}^{2}}{{n}_{{\mathrm{P}}}}\big)}^{2}}{{n}_{{\mathrm{P}}}-1}+\frac{{\big(\frac{{s}_{{\mathrm{N}}}^{2}}{{n}_{{\mathrm{N}}}}\big)}^{2}}{{n}_{{\mathrm{N}}}-1}}$$

We finally calculate the *p* value by:

4 $$p=\frac{1}{\sqrt{f\pi }}\frac{\Gamma \big(\frac{f+1}{2}\big)}{\Gamma \big(\frac{f}{2}\big)}\int _{-\infty }^{t}\frac{1}{{\big(1+\frac{{t}^{2}}{f}\big)}^{\frac{f+1}{2}}}dt$$

Where *t* is the *t*-score, *f* is the degree of freedom, Γ(.) is the Gamma function, and *p* is the probability that a single observation from the *t* distribution with *f* degrees of freedom will fall in the interval [–∞, *t*]. In other terms, the *p* is the probability to have by chance any *t*-score that is equal or below to the *t*-score *t*. Thus, the lower is the *p* value *p*, the higher is the significance of the *t*-score *t*, and consequently the higher is the separation between the two distributions of frequencies in the positive and the negative selections. We filtered the *p* values using a stringent cut-off of 10^−10^ to identify highly specific Ab clones. To complement the *p* values, we calculated Cohen’s *d* effect size coefficient^85^ to evaluate the difference between the means of the frequencies in the positive and the negative selections, and we kept *p* values with huge effect size^86^ (*d* > 2).

5 $$d=\frac{{\bar{x}}_{{\mathrm{P}}}-{\bar{x}}_{{\mathrm{N}}}}{\sqrt{\frac{({n}_{{\mathrm{P}}}-1){s}_{{\mathrm{P}}}^{2}+({n}_{{\mathrm{N}}}-1){s}_{{\mathrm{N}}}^{2}}{{n}_{{\mathrm{P}}}+{n}_{{\mathrm{N}}}-2}}}$$

### Statistics and reproducibility

The Welch–Satterthwaite version of the Student’s *t*-test in conjunction with the rank transformation was used for scoring specificity of candidate Ab clones for the targeted antigens^51^. The statistical significance of each *t*-score was evaluated by calculating the probability to see by chance any *t*-score below or equal the calculated *t*-score. Sample sizes are included in each figure legend. Individual data points are plotted where applicable.

### Reporting Summary

Further information on research design is available in the Nature Research Reporting Summary linked to this article.

## Supplementary information

Supplementary Information

Description of Supplementary Files

Supplementary Data 1

Supplementary Data 2

Supplementary Data 3

Supplementary Data 4

Supplementary Data 5

Supplementary Data 6

Reporting Summary

## Data Availability

The data and the results supporting the findings of this study are available within the paper and its Supplementary Data files 1–6. The complete NGS data presented in this work is publicly available at Zenodo repository^87^. Similarly, any additional data related to this study are available from the corresponding author upon reasonable request.

## Code availability

The C++ implementation of CellectSeq motif-based antibody discovery algorithm^87,88^ is publicly available at GitHub platform under the GNU GENERAL PUBLIC LICENSE. Any additional material and information related to this study is available from the corresponding author upon reasonable request.
