# Supplementary material for: CellectSeq: In silico discovery of antibodies targeting integral membrane proteins combining in situ selections and next-generation sequencing
Source: Commun Biol. 2021 May 12;4:561. doi: 10.1038/s42003-021-02066-5 (PMC8115320; doi:10.1038/s42003-021-02066-5)
Supplement: Supplementary file 1 — Supplementary Information [file 42003_2021_2066_MOESM1_ESM.pdf]

Table S1. Validation summary of CD151 Ab clones derived from CellectSeq

The antibody sequences that were predicted before and after filtering were synthesized as Fab protein and assayed for cellular binding on HEK293T-CD151+ cells via flow-cytometry. In situ validation result “Pass” = fluorescence signal 3-fold or greater than background (HEK293T-CD151- cells). Columns definition as inTable 1.

Selection	ID	Counts	%Total	Log10 P/N	L3								H1						H2										H3																			*Pvalue*	In situ valid
					107	108	109	110	113	114	115	116	30	35	36	37	38	39	55	56	57	58	59	62	63	64	65	66	107	108	109	110	111	111.1	111.2	111.3	111.4	111.5	112.5	112.4	112.3	112.2	112.1	112	113	114	115		
After filtering	CD151-1	226048	3.0E-01	2.17	S	F	F	-	-	-	-	P	I	L	S	Y	Y	S	M	S	I	Y	P	S	Y	G	Y	T	Y	S	H	Y	G	V	-	-	-	-	-	-	-	-	-	W	Y	G	A	2.2E-303	PASS
	CD151-2	137	1.8E-05	1.26	S	W	V	Y	-	-	S	L	I	I	Y	Y	Y	S	M	S	I	S	P	Y	S	G	Y	T	Y	S	P	Y	A	V	-	-	-	-	-	-	-	-	-	-	F	Y	G	8.0E-130	PASS
	CD151-3	74	9.8E-06	1.72	S	G	W	P	-	-	F	L	I	L	S	Y	Y	Y	M	S	I	Y	P	Y	Y	G	Y	T	Y	S	H	Y	G	V	-	-	-	-	-	-	-	-	-	W	Y	G	A	7.9E-294	PASS
	CD151-4	7	9.3E-07	0.54	S	S	Y	-	-	-	S	L	I	L	S	Y	S	Y	M	S	I	S	S	S	Y	G	Y	T	S	S	Y	S	G	-	-	-	-	-	-	-	-	-	-	-	-	G	A	3.1E-24	PASS
Before filtering	CD151-5	361	4E-05	3.6	S	F	F	-	-	-	P	I	I	S	Y	S	S	M	S	I	S	P	Y	S	G	S	T	Y	S	Y	-	-	-	-	-	-	-	-	-	-	-	-	-	-	-	A	L	<1E-200	FAIL
	CD151-6	2869	3E-04	4.9	S	F	F	-	-	-	P	I	I	Y	S	S	S	I	S	I	S	P	S	S	G	Y	T	Y	A	W	G	H	-	-	-	-	-	-	-	-	-	-	-	W	Y	G	F	<1E-200	FAIL
	CD151-7	38498	5E-03	5.0	S	F	F	-	-	-	P	I	L	Y	Y	Y	Y	M	S	I	S	P	S	Y	G	S	T	Y	S	Y	G	P	-	-	-	-	-	-	-	-	-	-	G	Y	W	G	F	<1E-200	FAIL
	CD151-8	682	9E-05	6.7	S	F	F	-	-	-	P	I	I	Y	S	S	S	M	S	I	Y	P	S	Y	S	Y	T	S	G	S	G	-	-	-	-	-	-	-	-	-	-	-	-	-	Y	A	M	<1E-200	FAIL
	CD151-9	401	5E-05	4.1	S	F	F	-	-	-	P	I	L	Y	Y	S	S	M	S	I	S	P	S	Y	G	Y	T	S	A	A	G	Y	P	H	Y	S	-	-	-	W	S	Y	Y	Y	G	A	L	<1E-200	FAIL
	CD151-10	9760	1E-03	5.7	S	F	F	-	-	-	P	I	I	Y	S	S	S	I	S	I	Y	S	Y	Y	G	S	T	Y	G	P	W	-	-	-	-	-	-	-	-	-	-	-	-	-	W	A	F	<1E-200	FAIL
	CD151-11	400	5E-05	5.3	S	F	F	-	-	-	P	I	I	S	Y	Y	S	M	S	I	S	S	Y	Y	S	Y	T	Y	A	A	Y	P	Y	G	-	-	-	-	-	-	W	Y	S	G	G	A	F	<1E-200	FAIL
	CD151-12	127	1E-05	6.2	V	G	W	P	P	I	P	P	L	S	Y	Y	S	M	S	I	Y	P	S	Y	G	Y	T	Y	S	H	Y	G	V	-	-	-	-	-	-	-	-	-	W	Y	G	A	M	<1E-200	FAIL
	CD151-13	10626	1E-03	5.5	S	F	F	-	-	-	P	I	I	Y	S	Y	Y	I	S	I	S	S	Y	Y	S	S	T	Y	G	P	F	S	-	-	-	-	-	-	-	-	-	-	H	P	Y	G	F	<1E-200	FAIL
	CD151-14	111	1E-05	7.1	S	F	F	-	-	-	P	I	L	S	Y	Y	S	M	S	I	Y	P	S	Y	G	Y	T	Y	S	H	Y	G	V	W	-	-	-	-	-	-	-	Y	G	A	Y	G	L	<1E-200	FAIL
	CD151-15	306	4E-05	5.7	S	F	F	-	-	-	P	I	I	S	Y	S	S	I	S	I	Y	P	Y	Y	G	Y	T	S	S	P	Y	-	-	-	-	-	-	-	-	-	-	-	-	Y	H	A	M	<1E-200	FAIL
	CD151-16	338	4E-05	5.9	Y	Y	Y	P	A	V	P	I	L	S	Y	Y	S	M	S	I	Y	P	S	Y	G	Y	T	Y	S	H	Y	G	V	-	-	-	-	-	-	-	-	-	W	Y	G	A	M	<2E-038	FAIL
	CD151-17	1714	2E-04	5.6	S	F	F	-	-	-	P	I	I	S	Y	Y	S	M	S	I	Y	P	Y	Y	G	Y	T	S	S	S	Y	-	-	-	-	-	-	-	-	-	-	-	-	Y	H	A	M	<1E-200	FAIL
	CD151-18	92	1E-05	5.8	S	F	F	-	-	-	P	I	I	Y	S	S	S	I	Y	I	Y	S	Y	Y	G	S	T	Y	G	P	W	-	-	-	-	-	-	-	-	-	-	-	-	-	W	A	F	<1E-200	FAIL
	CD151-19	267	3E-05	4.6	S	F	F	-	-	-	P	I	I	S	Y	Y	S	M	S	I	S	S	Y	Y	G	S	T	S	S	W	S	F	S	W	G	-	-	-	-	-	P	G	G	G	Y	A	M	<1E-200	FAIL
	CD151-20	2620	3E-04	5.2	S	F	F	-	-	-	P	I	I	S	Y	Y	Y	I	S	I	Y	P	S	S	G	Y	T	Y	Y	S	S	-	-	-	-	-	-	-	-	-	-	-	-	-	H	G	L	<1E-200	FAIL
	CD151-21	3450	4E-04	5.5	S	F	F	-	-	-	P	I	F	S	S	S	S	I	S	I	Y	P	Y	S	G	Y	T	S	G	A	V	V	H	S	Y	G	G	Y	A	Y	H	H	S	Y	Y	G	L	<1E-200	FAIL
	CD151-22	862	1E-04	5.4	S	F	F	-	-	-	P	I	I	S	Y	Y	S	M	S	I	Y	P	Y	S	G	Y	T	Y	V	G	Y	W	-	-	-	-	-	-	-	-	-	-	-	G	Y	G	M	<1E-200	FAIL
	CD151-23	450	5E-05	7.2	S	F	F	-	-	-	P	I	L	S	Y	Y	S	M	S	I	Y	P	S	Y	G	Y	T	Y	F	H	Y	G	V	-	-	-	-	-	-	-	-	-	W	Y	G	A	M	<1E-200	FAIL
	CD151-24	1475	1E-04	4.0	S	F	F	-	-	-	P	I	L	S	Y	Y	S	M	S	I	Y	P	S	Y	G	Y	T	Y	G	P	W	H	G	-	-	-	-	-	-	-	-	-	-	S	Y	G	L	<1E-200	FAIL
	CD151-25	1313	1E-04	7.2	S	F	F	-	-	-	P	I	L	S	Y	Y	S	M	S	I	Y	P	S	Y	G	Y	T	Y	P	H	Y	G	V	-	-	-	-	-	-	-	-	-	W	Y	G	A	M	<1E-200	FAIL
	CD151-26	4820	6E-04	5.0	S	F	F	-	-	-	P	I	L	S	Y	Y	S	M	S	I	Y	P	S	Y	G	Y	T	Y	S	F	A	G	W	-	-	-	-	-	-	-	-	-	-	A	S	Y	A	F	<1E-200	FAIL
	CD151-27	46985	6E-03	5.2	S	F	F	-	-	-	P	I	L	S	Y	S	Y	M	S	I	Y	P	Y	S	G	S	T	Y	S	G	Y	W	G	-	-	-	-	-	-	-	-	-	-	-	A	Y	G	M	<1E-200	FAIL

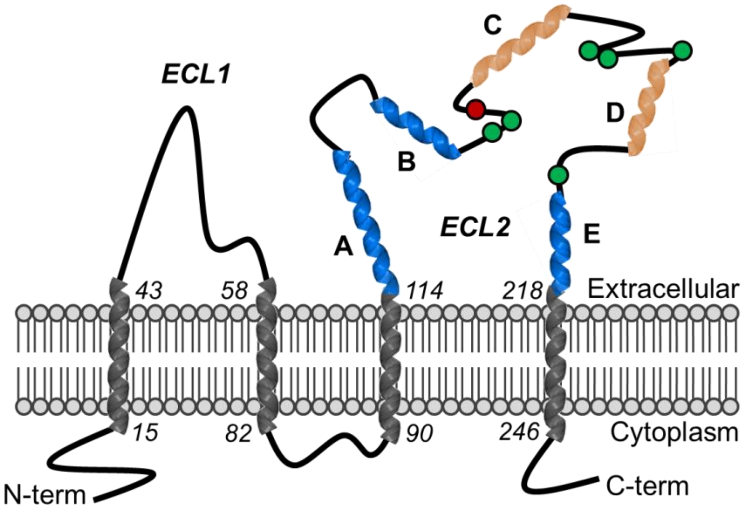

Figure S1. Schematic of CD151 structure

CD151 consists of four alpha-helical transmembrane domains, two extracellular loops one short (ECL1) and one long typically 100 amino acid residues (ECL2), and one very short intracellular loop, all flanked by relatively short cytoplasmic N-terminal and C-terminal tails. The EC1 loop displays small stature and low structural organization. The ECL2 loop is composed of five α-helical domains A, B, C, D, and E, forming stalk and head elements of a mushroom-like structure. The A, B and E helices (blue) are forming the constant region and are suggested to mediate homodimerization, while the C and D helices (orange) are forming the variable region and their flanking sequences mediate interactions with other proteins. The six cysteine residues in ECL2 are indicated in green, and the N-glycosylation site in red. The numbering represents the amino acid occurring before or after each transmembrane domain.

A

Name	Library-F Parental CDR NA Sequences	Length
L3	5'- **TCTTCTTATTCTCTGATC** -3'	18
H1	5'- **TTTTCTTCTTCTTCTATA** -3'	18
H2	5'- **TCTATTTCTTCTTCTTATGGCTATACTTAT** -3'	30
H3	5'- **ACTGTTCGTGGATCCAAAAAACCGTACTTCTCTGGTTGGGCTATG** -3'	45

B

Name	Library-F Variable CDR NA Sequences	Length
L3	5'- **X(3-7)CYGWTC** -3'	15 - 27
H1	5'- **MTCTMTTMTTMTTMTATS** -3'	18
H2	5'- **TMTATTTMTYCTTMTTMTRGCTMTACTTMT** -3'	30
H3	5'- **X(1-17)GSTWTK** -3'	9 - 57

C

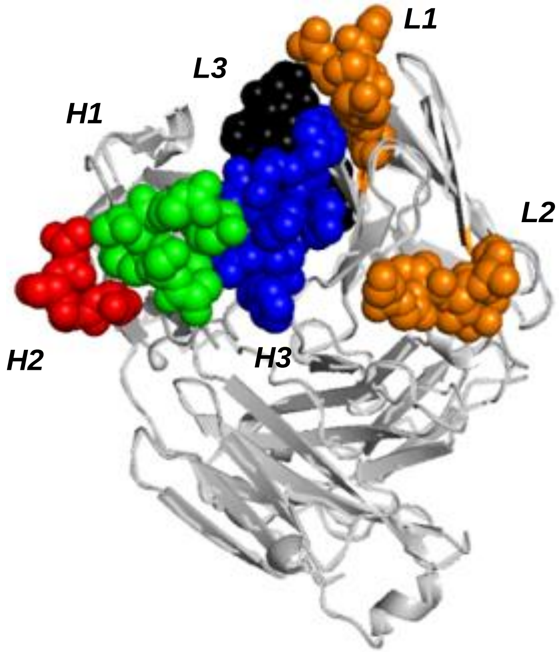

D

CDR-L3

107	108	109	114	115	116
X	X	X	X	PL	FI

CDR-H1

30	35	36	37	38	39
IL	YS	YS	YS	YS	IM

CDR-H2

55	56	57	58	59	62	63	64	65	66
YS	I	YS	PS	YS	YS	GS	YS	T	YS

CDR-H3

107	108	109	110	111	112	113	114	115
X	X	X	X	X	X	X	AG	FILM

Theoretical Diversity	
CDR-H1	64
CDR-H2	256
CDR-H3	7.5E+16
CDR-L3	1.7E+09

Figure S2. Library F CDR sequences

(A) Nucleotide sequences are formatted according to IUPAC code, and showing the nucleotide composition of template (parental) CDR sequences utilized to construct library F. Among 3x1010 unique clones in Library F, diversity was incorporated in approximately 80% of the population in each CDR and the retention of template sequences in the remainders. **(B)** Composition of pattern (variable) nucleotide sequences in library F, where the pattern CDR sequences describe the composition and length diversity introduced to CDRs-H1, H2, H3 and L3 by allowing loop lengths that are found within these regions of natural antibodies. X(3-7) and X(1-17) indicates the insertion of 3 to 7 and 1 to 17 tri-nucleotides from a mixture designed to contain nine different amino acids of the following composition; 25% Tyr, 20% Ser, 20% Gly, 10% Ala, and 5% each of Phe, Trp, His, Pro and Val, all respectively. **(C)** Framework Structure of Fab region showing the CDR loops L1 and L2 (orange), L3 (black), H1 (green), H2 (red), and H3 (blue) as spheres. The figure was generated using PyMOL (http://www.pymol.org/) with crystal structure coordinates (Protein Data Bank entry 1MIM). **(D)** Description of the synthetic antibody library F CDR amino acid sequences, highlighting the CDR diversity by position (shaded in gray are fixed positions). Allowed amino acids are denoted by the single-letter code, where X denotes a mixture of nine amino acids (Y, S, G, A, F, W, H, P or V). The lengths of CDR-L3 and CDR-H3 may vary from 3–9 and 1–19 respectively and the residue numbering is according to the IMGT scheme. The theoretical diversity (all possible amino acid compositions) of each CDR sequence is displayed.

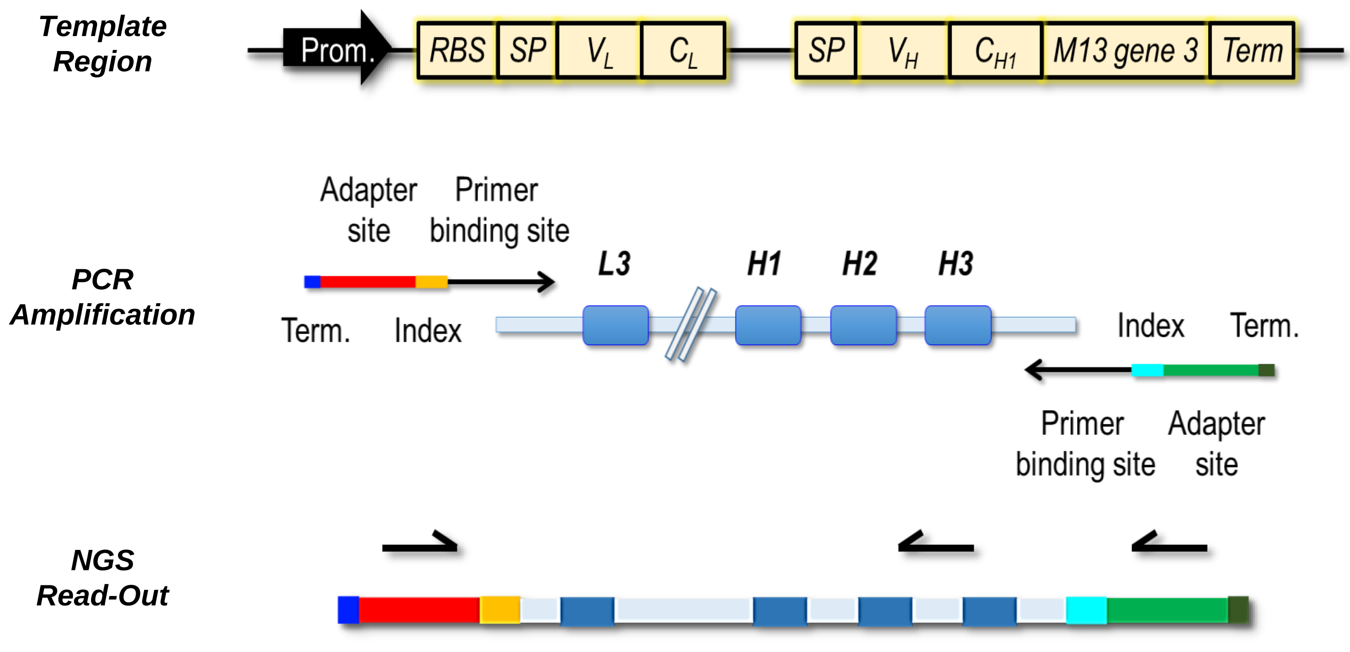

Figure S3. Description of NGS strategy for Illumina read-out of the diversified CDRs
The template DNA Fab region includes a PhoA promoter followed by a ribosome binding site (RBS) and two gene cassettes with signal peptide (SP) sequences and light or heavy antibody regions followed by the M13 gene-3 for display on phage particles. The phagemid template Fab region is PCR amplified utilizing two distinct primers that contain barcoded indexes and Illumina adapter sites. The amplicons are sequenced with three distinct read out primers that cover the sequences of the diversified regions L3, H1, H2, and H3.

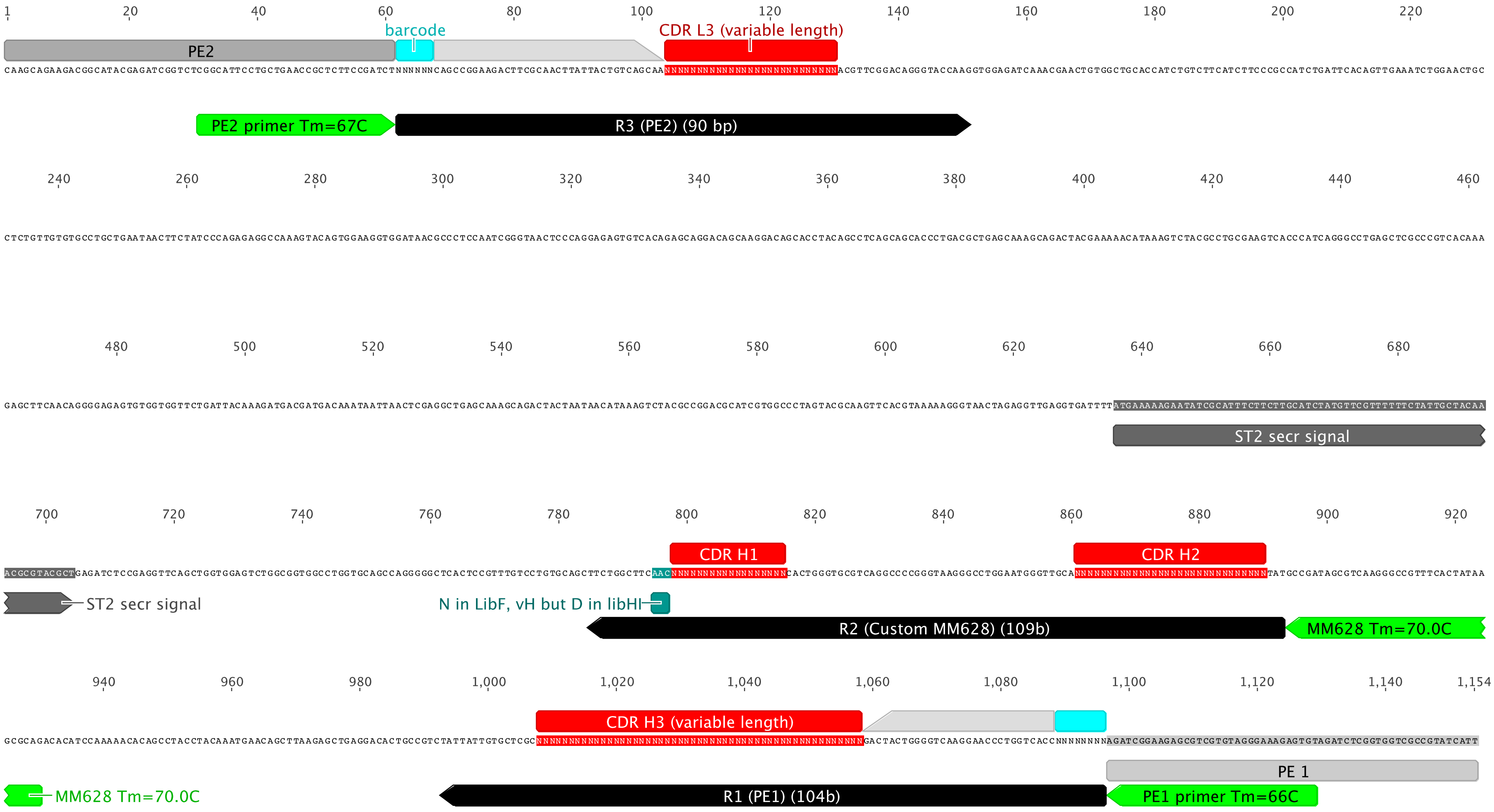

Figure S4. Library F NGS three-read diagram

Library F NGS three-read diagram (R1, R2, and R3) showing annealing regions of primers (PE1, PE2, and Custom MM628) on Ab framework.

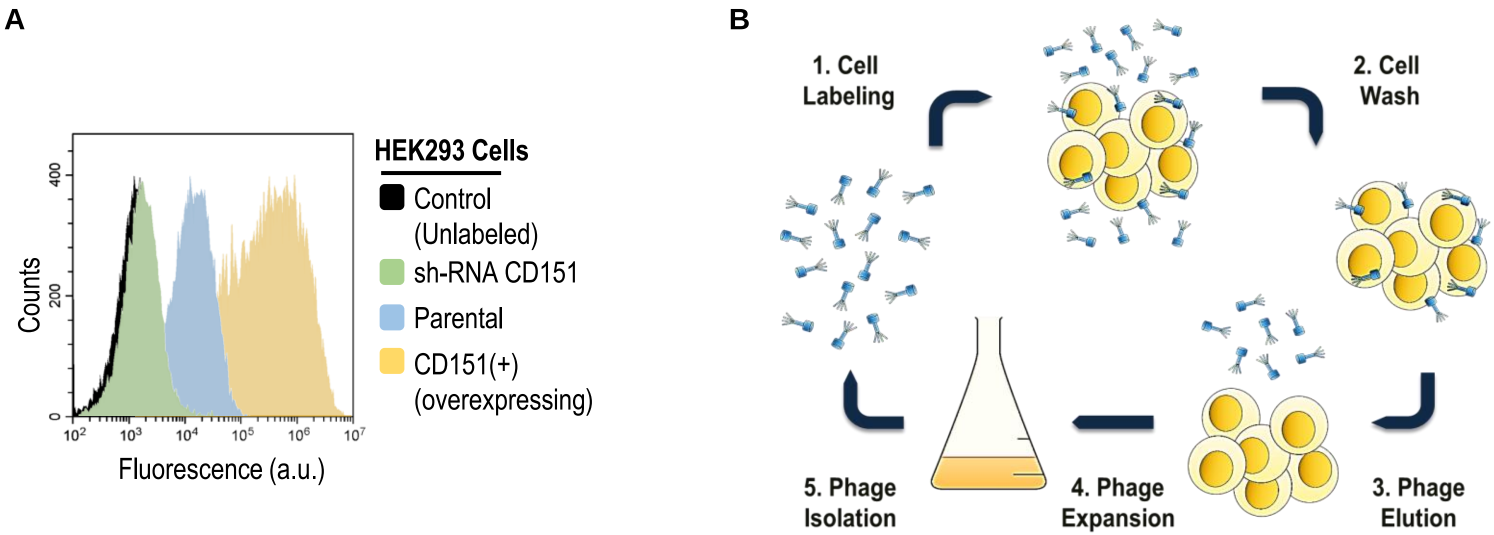

Figure S5. Validation of CD151 cell lines and schematic of round of selection

(A) Flow-cytometry histograms showing the CD151 surface expression of different HEK293T cell lines. Measurements were performed using an overexpressing CD151 cell line (HEK293T-CD151+), a short hair-pin RNA CD151 knockdown cell line(HEK293T-CD151-) cell line, and the parental HEK293T cell line, and CD151 surface expression measured utilizing a mouse anti-human CD151 PE conjugated IgG (Biolegend; cat. 350408). **(B)** Schematic of a round of selection where amplified Fab-phage is utilized to label antigens at the surface of live mammalian cells. After cellular wash and elution, the Fab-phage are amplified in E. coli and isolated for the next round of selection.

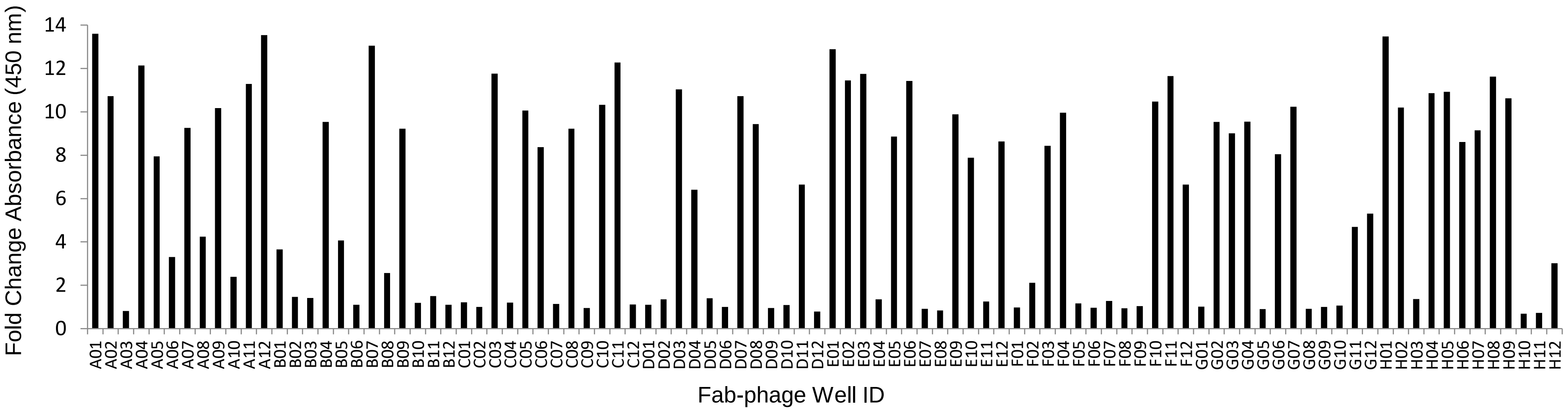

Figure S6. Validation summary of CD151 Ab clones derived from manual Ab screening
Binding measurements of Fab-phage to CD151 expressing cells (HEK293T-CD151+) by single-point cellular ELISA measurements. The fold change signal is measured by taking the ratio of signal from HEK293T-CD151+ cells over HEK293T-CD151- cells. A fold change signal of 5 or greater is deemed as a potential positive CD151 Fab-phage binder.

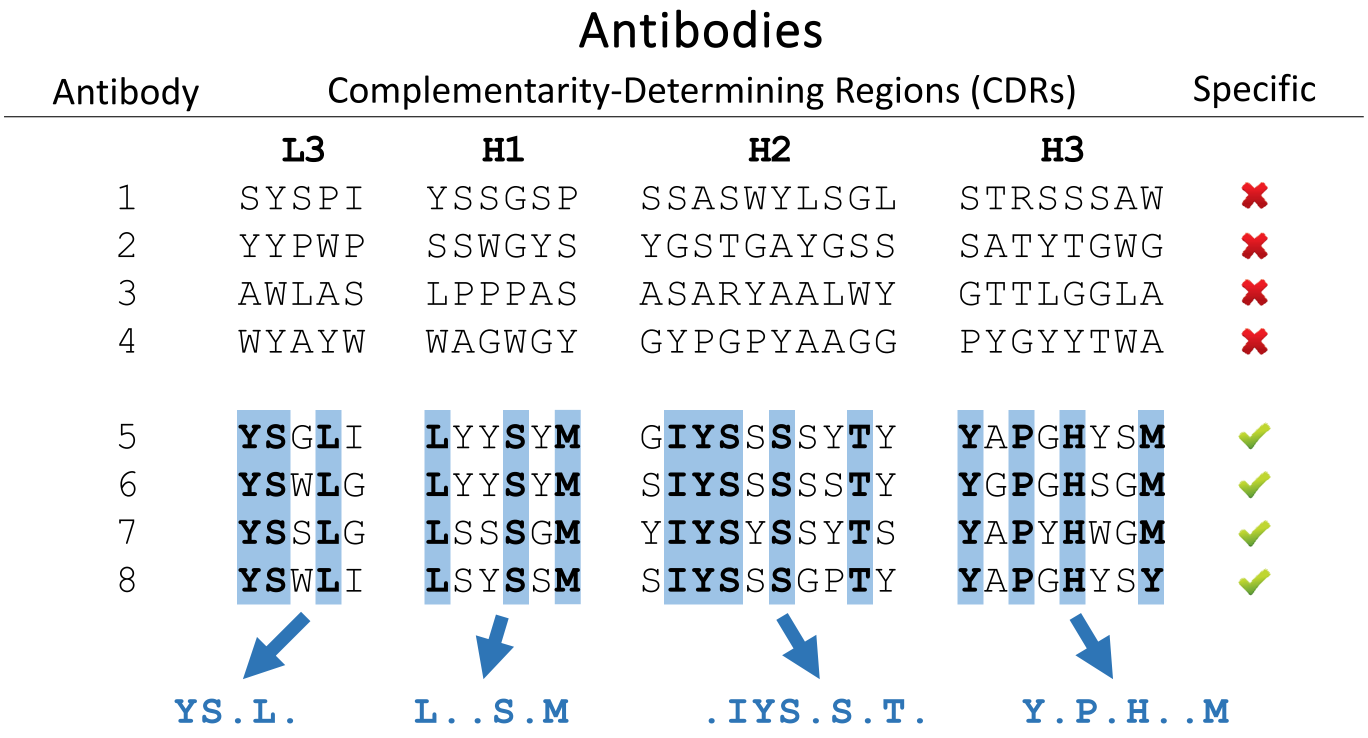

Figure S7. Linear information of paratope motifs as predictor for specificity of antibodies

Representation of the premise stating that highly selective Abs are enriched with paratope motifs that enable specific recognition of target epitopes, whereas non-specific Abs lack such enrichment.

A

CD151_HUMAN (100%), 28,295.3 Da
CD151 antigen OS=Homo sapiens GN=CD151 PE=1 SV=3
26 exclusive unique peptides, 39 exclusive unique spectra, 61 total spectra, 88/253 amino acids (35% coverage)

```
MGEFNEKKTT CGTVCLKYLL FTYNCCFWLA GLAVMAVGIW TLALKSDYIS
LLASGTYLAT AYILVVAGTV VMVTGVLGCC ATFKERRNLL RLYFILLLII
FLLEIIAGIL AYAYYQQLNT ELKENLKDTM TKRYHQPGHE AVTSAVDQLQ
QEFHCCGSNN SQDWRDSEWI RSQEAGGRVV PDSCCKTVVA LCGQRDHASN
IYKVEGGCIT KLETFIQEHL RVIGAVGIGI ACVQVFGMIF TCCLYRSLKL
EHY
```

Peptide Sequence	Prob	NTT	Observed	Actual Mass	Charge	Delta Da	Delta PPM	Retention Time	TIC	Start	Stop
(R)YHQPGHEAVTSAVD(Q)	100%	1	755.8512	1,509.69	2	0.002978	1.971	1,199.02	28,219.70	134	147
(R)YHQPGHEAVTSAVDqLQQEFHccGSNNSQDWR(D)	100%	2	1,262.88	3,785.62	3	0.02026	5.349	2,083.31	349181	134	165
(R)YHQPGHEAVTSAVDQLQQEFHccGSNNSQDWR(D)	100%	2	947.162	3,784.62	4	0.005595	1.478	2,091.73	679396	134	165
(R)YHQPGHEAVTSAVDQLQQEFHccGSNNSQDWR(D)	100%	2	757.9313	3,784.62	5	0.007019	1.854	2,044.05	2404360	134	165
(R)YHQPGHEAVTSAVDQLQQEFHccGSNNSQDWR(D)	100%	2	1,262.55	3,784.62	3	0.007071	1.868	2,084.86	242073	134	165
(R)YHQPGHEAVTSAVDqLQQEFHccGSNNSQDWR(D)	100%	2	947.4098	3,785.61	4	0.01278	3.375	2,110.59	749697	134	165
(R)YHQPGHEAVTSAVDQLQQEFHccGSNNSQDWR(D)	100%	2	947.1612	3,784.62	4	0.002595	0.6856	2,116.30	716182	134	165
(R)YHQPGHEAVTSAVDQLQQEFHccGSNNSQDWR(D)	100%	2	757.9298	3,784.61	5	-0.0006806	-0.1798	2,076.86	3128270	134	165
(R)YHQPGHEAVTSAVDQLQQEFHccGSNNSQDWR(D)	100%	2	947.1624	3,784.62	4	0.007195	1.901	2,061.91	2842120	134	165
(R)YHQPGHEAVTSAVDQLQQEFHccGSNNSQDWR(D)	100%	2	757.9311	3,784.62	5	0.005819	1.537	2,060.00	4550050	134	165
(R)YHQPGHEAVTSAVDQLqqEFHccGSNNSQDWR(D)	100%	2	758.3284	3,786.61	5	0.02414	6.373	2,131.28	692289	134	165
(R)YHQPGHEAVTSAVDQLQQEFHccGSNNSQDWR(D)	100%	2	947.1622	3,784.62	4	0.006515	1.721	2,045.04	1510660	134	165
(R)YHQPGHEAVTSAVDQLQQEFHccGSNNSQDWR(D)	100%	2	947.1636	3,784.63	4	0.012	3.169	2,078.74	1955760	134	165
(R)YHQPGHEAVTSAVDQLQQEFHccGSnNSQDWR(D)	100%	2	947.4146	3,785.63	4	0.03198	8.445	2,128.90	576662	134	165
(R)YHQPGHEAVTSAVDQLQqEFHccGSNNSQDWR(D)	100%	2	947.415	3,785.63	4	0.03374	8.91	2,108.86	657494	134	165
(R)YHqPGHEAVTSAVDQLQQEFHccGSNNSQDWR(D)	100%	2	758.1332	3,785.63	5	0.0323	8.531	2,110.18	1119500	134	165
(R)YHQPGHEAVTSAVDQLQQEFHccGSNNSQDWRDSEWIR(S)	100%	2	1,143.75	4,570.97	4	-0.009305	-2.035	2,216.99	498966	134	171
(R)YHQPGHEAVTSAVDQLQQEFHccGSNNSQDWRDSEWIR(S)	100%	2	1,143.75	4,570.98	4	0.003095	0.677	2,242.54	578642	134	171
(R)YHQPGHEAVTSAVDQLQQEFHccGSNNSQDWRDSEWIR(S)	100%	2	1,143.76	4,571.00	4	0.0255	5.576	2,220.05	369365	134	171
(R)YHQPGHEAVTSAVDQLQQEFHccGSNNSQDWRDSEWIR(S)	99%	2	1,143.75	4,570.99	4	0.008695	1.902	2,251.29	381205	134	171
(N)SQDWRDSEWIR(S)	100%	1	493.2322	1,476.67	3	0.000222	0.1502	1,798.72	549618	161	171
(R)DSEWIRSQEAGGR(V)	100%	2	497.5716	1,489.69	3	0.001902	1.276	1,376.11	329755	166	178
(R)DSEWIRSQEAGGR(V)	100%	2	745.8527	1,489.69	2	-0.000222	-0.1489	1,375.76	126385	166	178
(R)DSEWIRSQEAGGR(V)	100%	2	745.8544	1,489.69	2	0.003178	2.132	1,391.74	96,599.50	166	178
(R)DSEWIRSQEAGGR(V)	100%	2	497.5712	1,489.69	3	0.000612	0.4105	1,391.58	209671	166	178
(S)EWIRSQEAGGR(V)	100%	1	644.8229	1,287.63	2	-0.000822	-0.6379	1,380.40	184578	168	178
(E)WIRSQEAGGR(V)	100%	1	580.3019	1,158.59	2	-0.000142	-0.1225	1,378.08	102108	169	178
(W)IRSQEAGGR(V)	100%	1	487.2625	972.5103	2	0.000178	0.1828	1,378.79	83,011.00	170	178
(I)RSQEAGGR(V)	99%	1	430.7202	859.4258	2	-0.000262	-0.3045	1,383.36	97,702.20	171	178
(R)SQEAGGRVVPDSccK(T)	100%	2	825.3724	1,648.73	2	0.0004274	0.2591	1,056.88	99,868.40	172	186
(R)SQEAGGRVVPDSccK(T)	100%	2	550.5834	1,648.73	3	-0.001409	-0.8538	1,056.50	173209	172	186
(Q)EAGGRVVPDSccK(T)	100%	1	717.8268	1,433.64	2	-0.0002526	-0.1761	1,057.10	28,007.50	174	186
(E)AGGRVVPDSccK(T)	100%	1	653.3046	1,304.59	2	-0.002113	-1.618	1,059.11	25,656.50	175	186
(A)GGRVVPDSccK(T)	100%	1	617.7861	1,233.56	2	-0.001893	-1.533	1,060.63	21,933.20	176	186
(R)VVPDSccK(T)	99%	2	482.7147	963.4149	2	-0.0005526	-0.573	952.392	18,906.60	179	186
(R)VVPDSccKTVVAL(C)	100%	1	724.3693	1,446.72	2	0.002767	1.912	1,840.22	1079580	179	191
(R)VVPDSccKTVVALcGQR(D)	100%	2	974.9749	1,947.94	2	0.002182	1.12	1,550.36	186627	179	195
(R)VVPDSccKTVVALcGQR(D)	100%	2	650.318	1,947.93	3	-0.0007439	-0.3817	1,549.32	888369	179	195
(V)PDSccKTVVALcGQR(D)	100%	1	875.9058	1,749.80	2	0.0007821	0.4467	1,549.87	156243	181	195
(V)PDSccKTVVALcGQR(D)	100%	1	584.2719	1,749.79	3	-0.002394	-1.367	1,550.19	243015	181	195
(K)TVVALcGQR(D)	100%	2	502.2718	1,002.53	2	0.0009527	0.9493	1,386.86	1772130	187	195
(K)TVVALcGQR(D)	100%	2	502.2715	1,002.53	2	0.0002127	0.2119	1,419.50	136818	187	195
(K)TVVALcGQR(D)	100%	2	502.2712	1,002.53	2	-0.0002873	-0.2863	1,370.84	2156330	187	195
(K)TVVALcGQR(D)	100%	2	502.2717	1,002.53	2	0.0007727	0.77	1,403.70	221122	187	195
(K)TVVALcGQR(D)	100%	2	502.2717	1,002.53	2	0.0007127	0.7102	1,436.99	100389	187	195
(K)TVVALcGqR(D)	100%	2	502.7637	1,003.51	2	0.0006967	0.6936	1,541.87	125093	187	195
(K)TVVALcGQRDHASNIYK(V)	100%	2	966.4936	1,930.97	2	0.004213	2.181	1,352.36	43,553.40	187	203
(K)TVVALcGQRDHASNIYK(V)	100%	2	644.6626	1,930.97	3	-0.002463	-1.275	1,346.18	304195	187	203
(K)TVVALcGQRDHASNIYK(V)	99%	2	644.6635	1,930.97	3	0.0000867	0.04488	1,363.61	132470	187	203
(T)VVALcGQR(D)	99%	1	451.7477	901.4809	2	0.0004927	0.5459	1,374.79	100389	188	195
(R)DHASNIYK(V)	99%	2	474.2326	946.4506	2	-0.000222	-0.2343	932.124	9,219.32	196	203
(R)DHASNIYKVEGG(C)	100%	1	645.3095	1,288.60	2	-0.000422	-0.3272	1,251.10	56,776.00	196	207
(R)DHASNIYKVEGGcITK(L)	100%	2	896.4385	1,790.86	2	0.0001127	0.06289	1,346.51	68,887.70	196	211
(R)DHASNIYKVEGGcITK(L)	100%	2	896.4393	1,790.86	2	0.001773	0.9893	1,363.78	61,355.90	196	211
(R)DHASNIYKVEGGcITK(L)	100%	2	597.9607	1,790.86	3	-0.002063	-1.151	1,343.67	162827	196	211
(R)DHASNIYKVEGGcITK(L)	100%	2	597.9618	1,790.86	3	0.001237	0.6902	1,360.94	458754	196	211
(R)DHASNIYKVEGGcITK(L)	100%	2	448.7228	1,790.86	4	-0.0000793	-0.04426	1,357.48	104237	196	211
(K)LETFIQEHLR(V)	100%	2	429.234	1,284.68	3	-0.002598	-2.021	1,756.47	472965	212	221
(K)LETFIQEHLR(V)	100%	2	429.2353	1,284.68	3	0.001452	1.129	1,772.35	561276	212	221
(L)ETFIQEHLR(V)	100%	1	586.8072	1,171.60	2	0.001178	1.005	1,764.66	293604	213	221
(E)TFIQEHLR(V)	99%	1	522.2858	1,042.56	2	0.000978	0.9372	1,762.75	272648	214	221

B

CD151_HUMAN (100%), 28,295.3 Da
CD151 antigen OS=Homo sapiens GN=CD151 PE=1 SV=3
7 exclusive unique peptides, 9 exclusive unique spectra, 11 total spectra, 46/253 amino acids (18% coverage)

M G E F N E K K T T C G T V C L K Y L L F T Y N C C F W L A G L A V M A V G I W T L A L K S D Y I S
L L A S G T Y L A T A Y I L V V A G T V V M V T G V L G C C A T F K E R R N L L R L Y F I L L L I I
F L L E I I A G I L A Y A Y Y Q Q L N T E L K E N L K D T M T K R Y H Q P G H E A V T S A V D Q L Q
Q E F H C C G S N N S Q D W R **D S E W I** **R S Q E A G G R V V** **P D S C C K T V V A** **L C G Q R D H A S N**
I Y K V E G G C I T **K** L E T F I Q E H L R V I G A V G I G I A C V Q V F G M I F T C C L Y R S L K L
E H Y

Peptide Sequence	Prob	NTT	Observed	Actual Mass	Charge	Delta Da	Delta PPM	Retention Time	TIC	Start	Stop
(R)DSEWIRSQEAGGR(V)	100%	2	497.5722	1,489.69	3	0.003702	2.483	1,400.59	177088	166	178
(R)DSEWIRSQEAGGR(V)	99%	2	745.8537	1,489.69	2	0.001778	1.193	1,398.42	61,353.10	166	178
(R)SQEAGGRVVPDSccK(T)	100%	2	825.3735	1,648.73	2	0.002547	1.544	1,058.33	40,111.70	172	186
(R)SQEAGGRVVPDSccK(T)	100%	2	550.5833	1,648.73	3	-0.001979	-1.199	1,057.88	69,459.50	172	186
(Q)EAGGRVVPDSccK(T)	100%	1	717.8262	1,433.64	2	-0.001453	-1.013	1,059.00	15,537.70	174	186
(E)AGGRVVPDSccK(T)	100%	1	653.305	1,304.60	2	-0.001253	-0.9594	1,060.56	8,986.21	175	186
(V)PDSccKTVVALcGQR(D)	100%	1	875.9066	1,749.80	2	0.002382	1.361	1,557.96	57,952.40	181	195
(K)TVVALcGQR(D)	100%	2	502.2715	1,002.53	2	0.0004127	0.4112	1,409.23	234913	187	195
(K)TVVALcGQR(D)	100%	2	502.2712	1,002.53	2	-0.0002873	-0.2863	1,392.37	359208	187	195
(R)DHASNIYKVEGGcITK(L)	100%	2	597.9618	1,790.86	3	0.001237	0.6902	1,364.27	72,486.00	196	211
(R)DHASNIYKVEGGcITK(L)	100%	2	597.9621	1,790.86	3	0.002137	1.192	1,375.54	93,607.90	196	211

C

CD151_HUMAN (100%), 28,295.3 Da
CD151 antigen OS=Homo sapiens GN=CD151 PE=1 SV=3
3 exclusive unique peptides, 3 exclusive unique spectra, 3 total spectra, 27/253 amino acids (11% coverage)

MGEFNEKKTT CGTVCLKYLL FTYNCCFWLA GLAVMAVGIW TLALKSDYIS
LLASGTYLAT AYILVVAGTV VMVTGVLGCC ATFKERRNLL RLYFILLLII
FLLEIIAGIL AYAYYQQLNT ELKENLKDTM TKRYHQPGHE AVTSAVDQLQ
QEFHCCGSNN SQDWRDSEWI RSQEAGGR**VV** **PDSCCKTVVA** **LCGQR**DHASN
IYKVEGGCIT K**LETFIQEHL** **R**VIGAVGIGI ACVQVFGMIF TCCLYRSLKL
EHY

Peptide Sequence	Prob	NTT	Observed	Actual Mass	Charge	Delta Da	Delta PPM	Retention Time	TIC	Start	Stop
(R)VVPDSccK(T)	100%	2	482.7129	963.4112	2	-0.00427	-4.43	901.061	1125540	179	186
(K)TVVALcGQR(D)	100%	2	502.2692	1,002.52	2	-0.00424	-4.221	1,358.82	1.84E+07	187	195
(K)LETFIQEHLR(V)	100%	2	429.234	1,284.68	3	-0.00257	-2.001	1,787.19	2656250	212	221

D

CD151_HUMAN (100%), 28,295.3 Da
CD151 antigen OS=Homo sapiens GN=CD151 PE=1 SV=3
6 exclusive unique peptides, 10 exclusive unique spectra, 13 total spectra, 75/253 amino acids (30% coverage)

MGEFNEKKTT CGTVCLKYLL FTYNCCFWLA GLAVMAVGIW TLALKSDYIS
LLASGTYLAT AYILVVAGTV VMVTGVLGCC ATFKERRNLL RLYFILLLII
FLLEIIAGIL AYAYYQQLNT ELKENLKDTM TKR**YHQPGHE** **AVTSAVDQLQ**
QEFHCCGSNN **SQDWR**DSEWI RSQEAGGR**VV** **PDSCCKTVVA** **LCGQRDHASN**
IYKVEGGCIT **KLETFIQEHL** **R**VIGAVGIGI ACVQVFGMIF TCCLYRSLKL
EHY

Peptide Sequence	Prob	NTT	Observed	Actual Mass	Charge	Delta Da	Delta PPM	Retention Time	TIC	Start	Stop
(R)YHQPGHEAVTSAVDQLQQEFHccGSNNSQDWR(D)	100%	2	1,262.55	3,784.62	3	0.003983	1.052	1,970.22	2351090	134	165
(R)YHQPGHEAVTSAVDQLQQEFHccGSNNSQDWR(D)	100%	2	757.9265	3,784.60	5	-0.01718	-4.537	1,974.62	1.51E+07	134	165
(R)YHQPGHEAVTSAVDQLQQEFHccGSNNSQDWR(D)	100%	2	947.1572	3,784.60	4	-0.01379	-3.643	1,970.06	1.05E+07	134	165
(R)VVPDSccK(T)	100%	2	482.7151	963.4156	2	0.0001357	0.1407	978.921	299514	179	186
(K)TVVALcGQR(D)	100%	2	502.2713	1,002.53	2	-0.00009215	-0.09183	1,443.89	3.88E+07	187	195
(K)TVVALcGQR(D)	100%	2	502.2716	1,002.53	2	0.000542	0.5401	1,452.82	5224880	187	195
(K)TVVALcGQR(D)	100%	2	502.2712	1,002.53	2	-0.0002976	-0.2965	1,435.48	4.90E+07	187	195
(K)TVVALcGQR(D)	100%	2	502.2713	1,002.53	2	-0.0001389	-0.1384	1,471.77	2816750	187	195
(R)DHASNIYKVEGGcITK(L)	100%	2	896.4397	1,790.86	2	0.002476	1.382	1,357.90	1745420	196	211
(R)DHASNIYKVEGGcITK(L)	100%	2	597.9615	1,790.86	3	0.0003224	0.1799	1,356.12	5879890	196	211
(R)DHASNIYKVEGGcITK(L)	100%	2	448.7231	1,790.86	4	0.0008654	0.4829	1,359.21	5140110	196	211
(K)VEGGcITKLETFIQEHLR(V)	100%	2	710.7118	2,129.11	3	0.01933	9.073	2,351.10	5209280	204	221
(K)LETFIQEHLR(V)	100%	2	429.2356	1,284.68	3	0.002075	1.614	1,744.21	3.12E+07	212	221

E

CD151_HUMAN (100%), 28,295.3 Da
CD151 antigen OS=Homo sapiens GN=CD151 PE=1 SV=3
9 exclusive unique peptides, 12 exclusive unique spectra, 17 total spectra, 51/253 amino acids (20% coverage)

MGEFNEKKTT CGTVCLKYLL FTYNCCFWLA GLAVMAVGIW TLALKSDYIS
LLASGTYLAT AYILVVAGTV VMVTGVLGCC ATFKERRNLL RLYFILLLII
FLLEIIAGIL AYAYYQQLNT ELKENLKDTM TKRYHQPGHE AVTSAVDQLQ
QEFHCCGSNN **SQDWRDSEWI** **RSQEAGGRVV** **PDSCCKTVVA** **LCGQRDHASN**
IYKVEGGCIT **K**LETFIQEHL RVIGAVGIGI ACVQVFGMIF TCCLYRSLKL
EHY

Peptide Sequence	Prob	NTT	Observed	Actual Mass	Charge	Delta Da	Delta PPM	Retention Time	TIC	Start	Stop
(T)VVALcGQR(D)	100%	1	451.748	901.4815	2	0.001113	1.233	1,374.44	210971	188	195
(V)VALcGQR(D)	99%	1	402.2133	802.4121	2	0.0000527	0.06559	1,372.28	279206	189	195
(K)TVVALcGQRDHASNIYK(V)	100%	2	644.6629	1,930.97	3	-0.001563	-0.8092	1,341.91	186621	187	203
(K)TVVALcGQR(D)	100%	2	502.2721	1,002.53	2	0.001513	1.507	1,385.63	2584590	187	195
(K)TVVALcGQR(D)	100%	2	502.2711	1,002.53	2	-0.0004873	-0.4856	1,434.37	233961	187	195
(K)TVVALcGQR(D)	100%	2	502.2718	1,002.53	2	0.0009527	0.9493	1,369.27	2019500	187	195
(K)TVVALcGQR(D)	100%	2	502.2719	1,002.53	2	0.001073	1.069	1,416.82	255403	187	195
(K)TVVALcGQR(D)	100%	2	502.2714	1,002.53	2	0.0001127	0.1123	1,401.00	458732	187	195
(K)TVVALcGQR(D)	100%	2	502.2706	1,002.53	2	-0.001487	-1.482	1,467.31	128380	187	195
(R)SQEAGGRVVPDSccK(T)	100%	2	825.3726	1,648.73	2	0.0006674	0.4045	1,046.54	44,605.30	172	186
(R)SQEAGGRVVPDSccK(T)	100%	2	550.5833	1,648.73	3	-0.001829	-1.108	1,045.69	125102	172	186
(N)SQDWRDSEWIR(S)	100%	1	493.2327	1,476.68	3	0.001692	1.145	1,802.62	468069	161	171
(R)DSEWIRSQEAGGR(V)	100%	2	497.5711	1,489.69	3	0.000522	0.3502	1,376.61	259835	166	178
(R)DSEWIRSQEAGGR(V)	100%	2	745.8538	1,489.69	2	0.001978	1.327	1,374.75	133783	166	178
(R)DHASNIYKVEGGcITK(L)	100%	2	896.4396	1,790.86	2	0.002313	1.291	1,346.39	120239	196	211
(R)DHASNIYKVEGGcITK(L)	100%	2	597.9612	1,790.86	3	-0.0005633	-0.3144	1,343.34	267701	196	211
(R)DHASNIYK(V)	100%	2	474.2322	946.4499	2	-0.000942	-0.9942	920.784	18,269.90	196	203

F

CD151_HUMAN (100%), 28,295.3 Da
CD151 antigen OS=Homo sapiens GN=CD151 PE=1 SV=3
2 exclusive unique peptides, 2 exclusive unique spectra, 3 total spectra, 25/253 amino acids (10% coverage)

MGEFNEKKTT CGTVCLKYLL FTYNCCFWLA GLAVMAVGIW TLALKSDYIS
LLASGTYLAT AYILVVAGTV VMVTGVLGCC ATFKERRNLL RLYFILLLII
FLLEIIAGIL AYAYYQQLNT ELKENLKDTM TKRYHQPGHE AVTSAVDQLQ
QEFHCCGSNN SQDWRDSEWI RSQEAGGRVV PDSCCK**TVVA** **LCGQRDHASN**
IYKVEGGCIT **K**LETFIQEHL RVIGAVGIGI ACVQVFGMIF TCCLYRSLKL
EHY

Peptide Sequence	Prob	NTT	Observed	Actual Mass	Charge	Delta Da	Delta PPM	Retention Time	TIC	Start	Stop
(K)TVVALcGQR(D)	100%	2	502.2718	1,002.53	2	0.001013	1.009	1,370.38	302005	187	195
(K)TVVALcGQR(D)	100%	2	502.2702	1,002.53	2	-0.002227	-2.219	1,385.96	168863	187	195
(R)DHASNIYKVEGGcITK(L)	100%	2	597.961	1,790.86	3	-0.001163	-0.6492	1,341.86	192378	196	211

G

CD151_HUMAN (100%), 28,295.3 Da
CD151 antigen OS=Homo sapiens GN=CD151 PE=1 SV=3
5 exclusive unique peptides, 5 exclusive unique spectra, 16 total spectra, 43/253 amino acids (17% coverage)

```
MGEFNEKKTT CGTVCLKYLL FTYNCCFWLA GLAVMAVGIW TLALKSDYIS
LLASGTYLAT AYILVVAGTV VMVTGVLGCC ATFKERRNLL RLYFILLLII
FLLEIIAGIL AYAYYQQLNT ELKENLKDTM TKRYHQPGHE AVTSAVDQLQ
QEFHCCGSNN SQDWRDSEWI RSQEAGGRVV PDSCCKTVVA LCGQRDHASN
IYKVEGGCIT KLETFIQEHL RVIGAVGIGI ACVQVFGMIF TCCLYRSLKL
EHY
```

Peptide Sequence	Prob	NTT	Observed	Actual Mass	Charge	Delta Da	Delta PPM	Retention Time	TIC	Start	Stop
(R)VVPDSccK(T)	100%	2	482.7137	963.4129	2	-0.002563	-2.658	722.625	398406	179	186
(R)VVPDSccK(T)	100%	2	482.714	963.4134	2	-0.002075	-2.151	727.597	1345210	179	186
(R)VVPDSccK(T)	99%	2	482.7141	963.4136	2	-0.001892	-1.961	732.873	715476	179	186
(K)TVVALcGQR(D)	100%	2	502.2703	1,002.53	2	-0.002161	-2.153	1,435.08	3872810	187	195
(K)TVVALcGQR(D)	100%	2	502.2703	1,002.53	2	-0.002161	-2.153	1,414.72	5.56E+07	187	195
(K)TVVALcGQR(D)	99%	2	502.2703	1,002.53	2	-0.002039	-2.031	1,420.11	1.47E+07	187	195
(K)TVVALcGQR(D)	99%	2	502.2704	1,002.53	2	-0.001794	-1.788	1,409.30	6.29E+07	187	195
(K)TVVALcGQR(D)	99%	2	502.2705	1,002.53	2	-0.001611	-1.606	1,426.83	5096410	187	195
(K)TVVALcGQR(D)	96%	2	502.2702	1,002.53	2	-0.002344	-2.335	1,403.95	9753460	187	195
(R)DHASNIYK(V)	100%	2	474.2318	946.4489	2	-0.001922	-2.028	643.867	537703	196	203
(R)DHASNIYK(V)	99%	2	474.2317	946.4489	2	-0.001983	-2.093	649.119	893082	196	203
(R)DHASNIYKVEGGcITK(L)	100%	2	597.9601	1,790.86	3	-0.003732	-2.083	1,286.70	327999	196	211
(R)DHASNIYKVEGGcITK(L)	100%	2	597.9612	1,790.86	3	-0.0006196	-0.3458	1,292.41	222183	196	211
(R)DHASNIYKVEGGcITK(L)	100%	2	597.9598	1,790.86	3	-0.004648	-2.594	1,280.14	179594	196	211
(K)LETFIQEHLR(V)	100%	2	429.234	1,284.68	3	-0.002481	-1.93	1,762.13	3698930	212	221
(K)LETFIQEHLR(V)	99%	2	429.234	1,284.68	3	-0.002573	-2.001	1,768.77	2047600	212	221

H

CD151_HUMAN (95%), 28,295.3 Da
CD151 antigen OS=Homo sapiens GN=CD151 PE=1 SV=3
1 exclusive unique peptides, 1 exclusive unique spectra, 1 total spectra, 9/253 amino acids (4% coverage)

```
MGEFNEKKTT CGTVCLKYLL FTYNCCFWLA GLAVMAVGIW TLALKSDYIS
LLASGTYLAT AYILVVAGTV VMVTGVLGCC ATFKERRNLL RLYFILLLII
FLLEIIAGIL AYAYYQQLNT ELKENLKDTM TKRYHQPGHE AVTSAVDQLQ
QEFHCCGSNN SQDWRDSEWI RSQEAGGRVV PDSCCKTVVA LCGQRDHASN
IYKVEGGCIT KLETFIQEHL RVIGAVGIGI ACVQVFGMIF TCCLYRSLKL
EHY
```

Peptide Sequence	Prob	NTT	Observed	Actual Mass	Charge	Delta Da	Delta PPM	Retention Time	TIC	Start	Stop
(K)TVVALcGQR(D)	98%	2	502.2704	1,002.53	2	-0.001855	-1.849	1,372.09	4044640	187	195

Figure S8. Corroboration of specificity for CD151 using IP-MS

IP-MS peptide coverage results of CD151 protein: (A) Fab CD151-1 assayed utilizing HEK293T-CD151+ cells; (B) Fab CD151-1 assayed utilizing HT-1080 cells; (C) Fab CD151-2 assayed utilizing HEK293T-CD151+; (D) Fab CD151-2 assayed utilizing HT-1080 cells; (E) Fab CD151-3 assayed utilizing HEK293T-CD151+ cells; (F) Fab CD151-3 assayed utilizing HT-1080 cells (G) Fab CD151-4 assayed utilizing HEK293T-CD151+ cells; and (H) Fab CD151-4 assayed utilizing HT-1080 cells. NTT - Number of termini consistent with the enzymatic cleavage or tryptic termini; Observed - Mass over charge (M/Z) of the parent or precursor ion measured by the mass spectrometer.; Actual Mass - Peptide mass in Dalton obtained by multiplying the charge to the subtraction of one proton from the observed M/Z; Charge - Peptide charge; Delta Da - (Actual Mass - Theoretical Peptide Mass) in Dalton, where the Theoretical Peptide Mass or Calculated peptide mass, is given by the sum of amino acid residue masses included in the peptide plus a water molecule; Delta PPM - (Actual Mass - Theoretical Peptide Mass) in PPM also referred to in the spectrum as the Parent error. It is calculated by dividing the delta mass expressed in Dalton by the Actual Mass and then multiplied by one million.; Retention Time - Measured in seconds, it is included in the table only if the information is listed in the peak list of the loaded data; TIC - MS/MS Total Ion Current; Start - Peptide start index; and Stop - Peptide stop index.

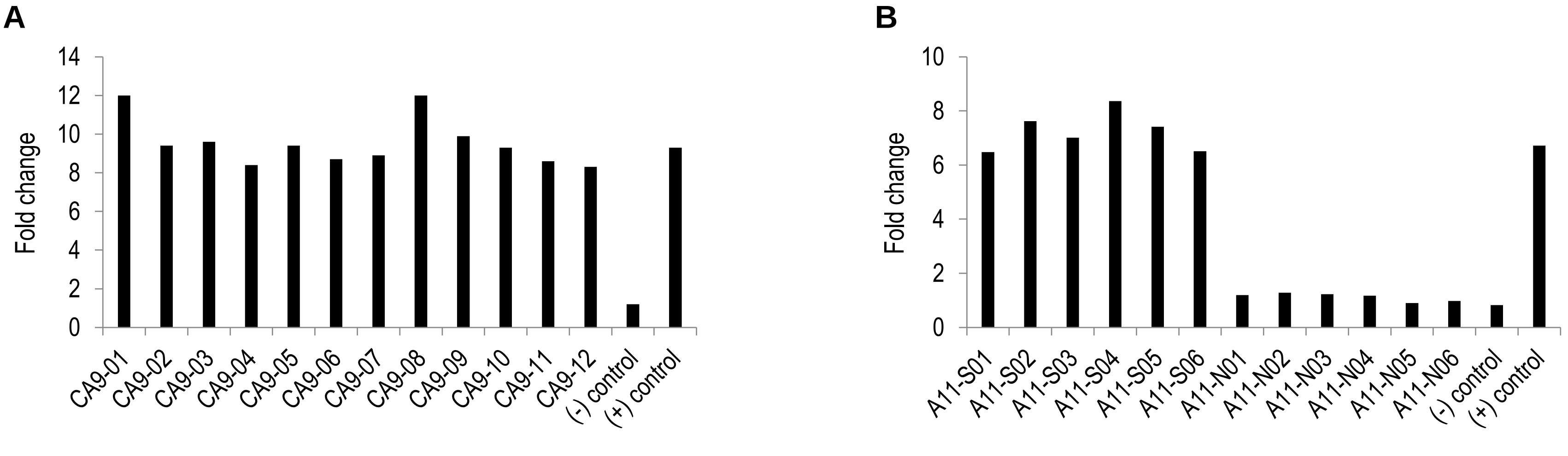

Figure S9. Validation of CellectSeq predicted clones against CA9 or integrin-α11 by cellular ELISA
(A) Bar graph showing fold change signal from Fab binding to HEKT293-CA9+ cells over parental HEKT293T-WT. **(B)** Bar graph showing fold change signal from Fab binding to C2C12-α11+ cells over parental C2C12-WT. For both assays, the x-axis indicates unique Fab clone assayed at 250 nM concentration, and a 5-fold or greater signal fold change indicates a positive unique clone binder.

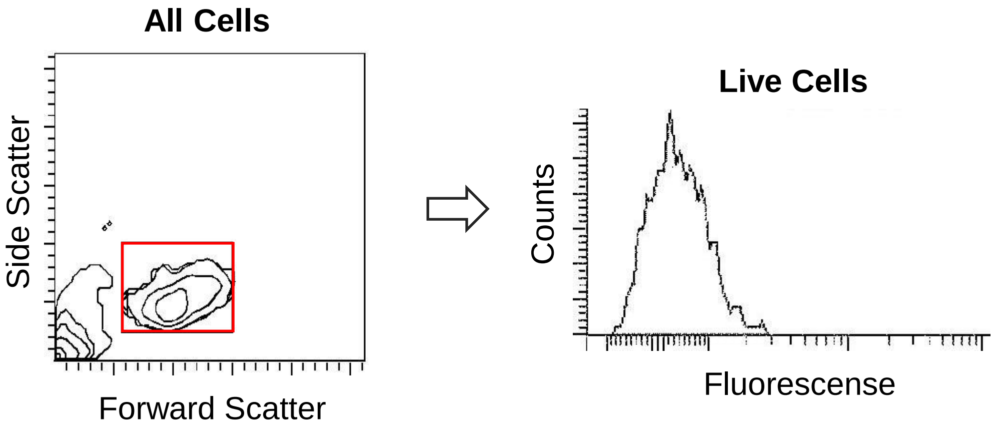

Figure S10. Description of flow-cytometry gating strategy.
The live cell population is gated from the “forward” and “side” scatter plot (red rectangle), and fluorescence measurements are determined from the live cell population (histogram).

A

Flow-cytometry Raw Data (MFI)

HEK293-CD151+ cells

Concentration (nM)	CD155-1	CD155-2	CD155-3	CD155-4
0.01	13829.5	4255.7	4523.3	4393.8
0.1	6412.3	-	-	-
0.5	19384.7	4430.8	4543.8	-
1	35850.4	4259.1	4751.2	27116.2
12.5	-	-	-	65651.3
25	509628.3	5502.9	9775.2	113213.4
50	774237.5	7599.9	13015.6	276488.9
100	895071.1	12275.3	20085.9	-
125	-	-	-	485336.3
250	-	-	-	719574.6
500	667600.2	15624.3	20428.8	915031.8
1000	-	14837.2	17823.4	-

HEK293-CD151- cells (control)

Concentration (nM)	CD155-1	CD155-2	CD155-3	CD155-4
0.01	3692.7	4613.8	4608.3	3961.9
0.1	3861.1	-	-	-
0.5	11766.4	4539.9	4101.3	-
1	38024	4483.2	4014	5019.8
12.5	-	-	-	5316.6
25	14366.3	4519.5	4116.6	6760.4
50	10824.1	4981.2	4736	6500.6
100	9499.3	5366.1	4746.3	-
125	-	-	-	14303.4
250	-	-	-	16308.3
500	8737.5	5815.2	4675.5	8226.5
1000	-	5335.8	4939.5	-

Subst. Background

Concentration (nM)	CD155-1	CD155-2	CD155-3	CD155-4
0.01	10136.8	358.1	85	431.9
0.1	2551.2	-	-	-
0.5	7618.3	109.1	442.5	-
1	2173.6	224.1	737.2	22096.4
12.5	-	-	-	60334.7
25	495262	983.4	5658.6	106453
50	763413.4	2618.7	8279.6	269988.3
100	885571.8	6909.2	15339.6	-
125	-	-	-	471032.9
250	-	-	-	703266.3
500	658862.7	9809.1	15753.3	906805.3
1000	-	9501.4	12883.9	-

Data Normalized

Concentration (nM)	CD155-1	CD155-2	CD155-3	CD155-4
0.01	0.011447	0.036507	0.005396	0.000476
0.1	0.002881	-	-	-
0.5	0.008603	0.011122	0.028089	-
1	0.002454	0.022846	0.046797	0.024367
12.5	-	-	-	0.066535
25	0.559257	0.100254	0.359201	0.117393
50	0.862057	0.266966	0.525579	0.297736
100	1	0.704366	0.973739	-
125	-	-	-	0.519442
250	-	-	-	0.775543
500	0.743997	1	1	1
1000	-	0.968631	0.817854	-

B

Flow-cytometry Raw Data (MFI)

	CD151-1	CD151-2	CD151-3	CD151-4
**Untreated**	250844.5	8082.9	12823.75	28273.5
**CD151-1**	18214.5	1679.05	1775.75	2685.7
**CD151-2**	18251.05	1407	1998.15	2659.2
**CD151-3**	72778.05	2452.65	3563.75	5938.25
**CD151-4**	36181.65	2225.5	2515.25	3425.5
**CD151 (-) cells**	10085.2	1268.4	2619.1	1495.2
**No Fab**	1500.05	1448.2	3367.65	2439.75

Data Normalized

	CD151-1	CD151-2	CD151-3	CD151-4
**Untreated**	100	100	100	100
**CD151-1**	7.261273	20.77287	13.84735	9.499001
**CD151-2**	7.275844	17.40712	15.58164	9.405273
**CD151-3**	29.01322	30.34369	27.79023	21.00288
**CD151-4**	14.42394	27.53343	19.614	12.11559
**CD151 (-) cells**	4.0205	15.69239	20.42382	5.288344
**No Fab**	0.598	17.91684	26.26104	8.629105

Figure S11. Raw data and analysis for Figure 5A and B panels.
(A) Raw data and analysis of panel A from Figure 5. Dose response for anti-CD151 Fabs assessed by flow cytometry fluorescence using HEK293T-CD151+ cells. The MFI signals were subtracted from background Fab binding to HEK293T-CD151- cells and normalized to the highest concentration value for each sample. **(B)** Raw data and analysis of panel B from Figure 5. Blocking of anti-CD151 Fabs binding to HEK293T-CD151+ cells by indicated IgGs, assessed by flow cytometry fluorescence, and then data normalized to the highest value of each group.
