# Supplementary material for: CellectSeq: In silico discovery of antibodies targeting integral membrane proteins combining in situ selections and next-generation sequencing
Source: Commun Biol. 2021 May 12;4:561. doi: 10.1038/s42003-021-02066-5 (PMC8115320; doi:10.1038/s42003-021-02066-5)
Supplement: Supplementary file 2 — Description of Supplementary Files [file 42003_2021_2066_MOESM2_ESM.pdf]

Description of Additional Supplementary Files

File name: Supplementary Data 1

Description: CD151 positive pool.

File name: Supplementary Data 2

Description: CD151 negative pool.

File name: Supplementary Data 3

Description: CA9 positive pool.

File name: Supplementary Data 4

Description: CA9 negative pool.

File name: Supplementary Data 5

Description: Integrin-α11 positive pool.

File name: Supplementary Data 6

Description: Integrin-α11 negative pool.
