# Supplementary material for: CellectSeq: In silico discovery of antibodies targeting integral membrane proteins combining in situ selections and next-generation sequencing
Source: Commun Biol. 2021 May 12;4:561. doi: 10.1038/s42003-021-02066-5 (PMC8115320; doi:10.1038/s42003-021-02066-5)
Supplement: Supplementary file 9 — Reporting Summary [file 42003_2021_2066_MOESM9_ESM.pdf]

nature research

Corresponding author(s): Sachdev Sidhu

Last updated by author(s): Feb 2, 2021

Reporting Summary

Nature Research wishes to improve the reproducibility of the work that we publish. This form provides structure for consistency and transparency in reporting. For further information on Nature Research policies, see our Editorial Policies and the Editorial Policy Checklist.

Statistics

For all statistical analyses, confirm that the following items are present in the figure legend, table legend, main text, or Methods section.

n/a	Confirmed	
☐	☒	The exact sample size (*n*) for each experimental group/condition, given as a discrete number and unit of measurement
☐	☒	A statement on whether measurements were taken from distinct samples or whether the same sample was measured repeatedly
☐	☒	The statistical test(s) used AND whether they are one- or two-sided<br>*Only common tests should be described solely by name; describe more complex techniques in the Methods section.*
☒	☐	A description of all covariates tested
☐	☒	A description of any assumptions or corrections, such as tests of normality and adjustment for multiple comparisons
☐	☒	A full description of the statistical parameters including central tendency (e.g. means) or other basic estimates (e.g. regression coefficient) AND variation (e.g. standard deviation) or associated estimates of uncertainty (e.g. confidence intervals)
☐	☒	For null hypothesis testing, the test statistic (e.g. *F*, *t*, *r*) with confidence intervals, effect sizes, degrees of freedom and *P* value noted<br>*Give P values as exact values whenever suitable.*
☒	☐	For Bayesian analysis, information on the choice of priors and Markov chain Monte Carlo settings
☒	☐	For hierarchical and complex designs, identification of the appropriate level for tests and full reporting of outcomes
☐	☒	Estimates of effect sizes (e.g. Cohen's *d*, Pearson's *r*), indicating how they were calculated

Our web collection on statistics for biologists contains articles on many of the points above.

Software and code

Policy information about availability of computer code

Data collection	No external data was collected in this study.
Data analysis	No external software was used for data analysis in this study. As stated in the manuscript the algorithms used in this study are made available.

For manuscripts utilizing custom algorithms or software that are central to the research but not yet described in published literature, software must be made available to editors and reviewers. We strongly encourage code deposition in a community repository (e.g. GitHub). See the Nature Research guidelines for submitting code & software for further information.

Data

Policy information about availability of data

All manuscripts must include a data availability statement. This statement should provide the following information, where applicable:

- Accession codes, unique identifiers, or web links for publicly available datasets
- A list of figures that have associated raw data
- A description of any restrictions on data availability

The data supportting the findings of this study are available with the manuscript, and upon reasonable request from the corresponding author.

Field-specific reporting

Life sciences study design

All studies must disclose on these points even when the disclosure is negative.

Sample size	Sample size is determined as the number of NGS readouts of each sample.
Data exclusions	No data exclusion. The NGS reads with sequencing errors were identified but retained in the analysis. This is explained in the manuscript.
Replication	NGS reads were measured in duplicate samples per cohort.
Randomization	Not relevant to our study.
Blinding	Not relevant to our study.

Reporting for specific materials, systems and methods

We require information from authors about some types of materials, experimental systems and methods used in many studies. Here, indicate whether each material, system or method listed is relevant to your study. If you are not sure if a list item applies to your research, read the appropriate section before selecting a response.

Materials & experimental systems

n/a	Involved in the study
☐	☒ Antibodies
☐	☒ Eukaryotic cell lines
☒	☐ Palaeontology and archaeology
☒	☐ Animals and other organisms
☒	☐ Human research participants
☒	☐ Clinical data
☒	☐ Dual use research of concern

Methods

n/a	Involved in the study
☒	☐ ChIP-seq
☐	☒ Flow cytometry
☒	☐ MRI-based neuroimaging

Antibodies

Antibodies used	In-House Synthetic Phage-Displayed Antibodies
Validation	Validation for antibodies was described in this study.

Eukaryotic cell lines

Policy information about cell lines

Cell line source(s)	HEK293T cell line source ATCC (cat: CRL-3216), HT1080 cell line ATCC (cat: CCL-121)
Authentication	The cell lines were purchased from ATCC and not authenticated since this was already performed by vendor.
Mycoplasma contamination	All cell lines tested negative for mycoplasma.
Commonly misidentified lines (See ICLAC register)	None

Flow Cytometry

Plots

Confirm that:

- ☒ The axis labels state the marker and fluorochrome used (e.g. CD4-FITC).
- ☒ The axis scales are clearly visible. Include numbers along axes only for bottom left plot of group (a 'group' is an analysis of identical markers).
- ☒ All plots are contour plots with outliers or pseudocolor plots.
- ☒ A numerical value for number of cells or percentage (with statistics) is provided.

Methodology

Sample preparation	Described in Materials and Methods section
Instrument	CytoFLEX-S flow-cytometer (Beckman Coulter)
Software	FlowJo v10.2 Software (FlowJo, LLC)
Cell population abundance	The cells are monoclonal, only one cell population.
Gating strategy	The cells were analyzed using forward versus side scatter (FSC vs SSC), and live cells gated and analyzed for Alexa-488 fluorescence (using a 488-nm laser with 530/25 nm detection filter). The gating of live cells is a standard procedure for flow-cytometry.

☒ Tick this box to confirm that a figure exemplifying the gating strategy is provided in the Supplementary Information.
